# Investigating the replacement of carboxylates with carboxamides to modulate the safety and efficacy of platinum(II) thioether cyanide scavengers

**DOI:** 10.1093/toxsci/kfad119

**Published:** 2023-11-11

**Authors:** Matthew M Behymer, Huaping Mo, Naoaki Fujii, Vallabh Suresh, Ari S Arzumanian, Adriano Chan, Anjali K Nath, Robyn McCain, Calum A MacRae, Randall Peterson, Gerry R Boss, Vincent Jo Davisson, Gregory T Knipp

**Affiliations:** Department of Industrial and Molecular Pharmaceutics, Purdue University, West Lafayette, Indiana 47907, USA; Department of Medicinal Chemistry and Molecular Pharmacology, Purdue University, West Lafayette, Indiana 47907, USA; Department of Medicinal Chemistry and Molecular Pharmacology, Purdue University, West Lafayette, Indiana 47907, USA; Department of Medicinal Chemistry and Molecular Pharmacology, Purdue University, West Lafayette, Indiana 47907, USA; Department of Medicinal Chemistry and Molecular Pharmacology, Purdue University, West Lafayette, Indiana 47907, USA; Department of Medicine, University of California, San Diego, California 92093, USA; Department of Cardiology, Beth Israel Deaconess Medical Center, Boston, Massachusetts 02215, USA; Purdue Translational Pharmacology CTSI Core Facility, Purdue University, West Lafayette, Indiana, USA; Division of Cardiovascular Medicine, Brigham and Women’s Hospital, Boston, Massachusetts 02115, USA; Department of Pharmacology and Toxicology, College of Pharmacy, University of Utah, Salt Lake City, Utah 84112, USA; Department of Medicine, University of California, San Diego, California 92093, USA; Department of Medicinal Chemistry and Molecular Pharmacology, Purdue University, West Lafayette, Indiana 47907, USA; Department of Industrial and Molecular Pharmaceutics, Purdue University, West Lafayette, Indiana 47907, USA

**Keywords:** exposures, countermeasure, cyanide, pharmacokinetics, chemical threat, safety and efficacy

## Abstract

Cyanide represents a persistent threat for accidental or malicious misuse due to easy conversion into a toxic gas and access to large quantities through several industries. The high safety index of hydroxocobalamin is a cornerstone quality as a cyanide scavenger. Unfortunately, intravenous infusion of hydroxocobalamin limits the utility in a mass casualty setting. We previously reported platinum(II) [Pt(II)] complexes with *trans-directing* sulfur ligands as an efficacious alternative to hydroxocobalamin when delivered by a bolus intramuscular (IM) injection in mice and rabbits. Thus, to enable Pt(II) as an alternative to hydroxocobalamin, a high safety factor is needed. The objective is to maintain efficacy and mitigate the risk of nephrotoxicity. Platinum amino acid complexes with the ability to form 5- or 6-membered rings and possessing either carboxylates or carboxamides are evaluated *in vitro* for cyanide scavenging. *In vivo* efficacy *wa*s evaluated in the zebrafish and mice cyanide exposure models. In addition, Pt(II) complex toxicity and pharmacokinetics were evaluated in a cyanide naive Sprague Dawley model. Doses for toxicity are escalated to 5× from the efficacious dose in mice using a body surface area adjustment. The results show the carboxamide ligands display a time and pH dependence on cyanide scavenging *in vitro* and efficacy *in vivo*. Additionally, exchanging the carboxylate for carboxamide showed reduced indications of renal injury. A pharmacokinetic analysis of the larger bidentate complexes displayed rapid absorption by IM administration and having similar plasma exposure. These findings point to the importance of pH and ligand structures for methionine carboxamide complexes with Pt(II).

## Graphical Abstract

**Graphical Abstract:**
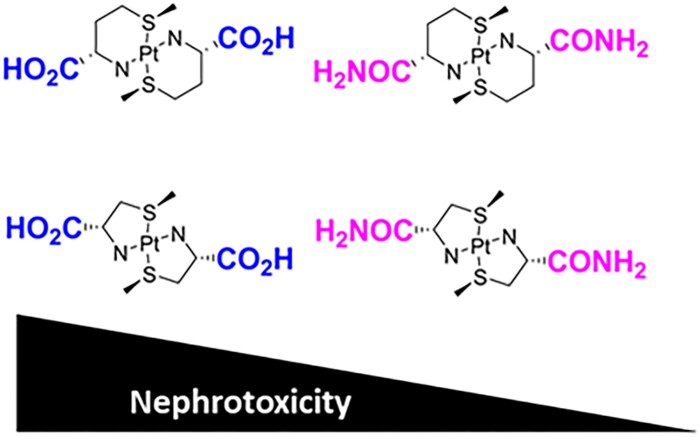

A leading cause of smoke inhalation-related deaths is suspected to be a result of cyanide poisoning (Anseeuw *et al.*, 2013; Baud *et al.*, 1991). The materials and products used in everyday household items can release cyanide when burned (Anseeuw *et al.*, 2013). Also, cyanide and cyanide derivatives are often used in many industries, such as metal polishing, mining, photographic development, and the chemical production of products like pesticides (Bhattacharya and Flora, 2015). In total, around 600 000 metric tons of cyanide are produced annually for industrial applications (Amizet *et al.*, 2011). Production of cyanide is also easily achieved, with synthesis requiring readily available ingredients such as ammonia and methane (Grabow *et al.*, 2011). In the presence of acid, hydrogen cyanide is volatile, filling the immediate air with deadly gas. Based on potential widespread availability and easy gas generation, cyanide presents itself as a threat for misuse to induce high mortality and morbidity. An example of misuse is best illustrated by the subway attacks in Tokyo, where terrorists placed cyanide salt and acid into trash cans attempting to fill the station with lethal gas that was averted by police intervention (Kristof, 1995). However, the incident at Jonestown in Guyana is a more tragic example of what can happen when access to cyanide is unchecked and used maliciously (Conroy, 2018).

Cyanide is a systemic poison that is known to inhibit cellular respiration by reversible inhibition of cytochrome c oxidase (Acute Exposure Guideline Levels for Selected Airborne Chemicals, 2002; Leavesley *et al.*, 2008). The fast action of cyanide on physiological functions requires rapid treatment options to reduce morbidity and mortality. Approximately 50% of mice exposed to constant atmospheric cyanide concentrations as low as 177 ppm died within 30 min of exposure (Alarie, 2002). Hydroxocobalamin is administered via intravenous infusion (IV) over approximately 15 min and is currently approved in the United States to treat cyanide poisoning (Shepherd and Velez, 2008). In some cases, a higher dose over a longer time is required, thus adding to the risks of comorbidities including drug-induced renal impairment (Shepherd and Velez, 2008). Evidence strongly supports that a prompt delivery of cyanide scavengers after exposure can significantly improve survival rates (Thompson *et al.*, 2019).

In the scenario of a potential mass casualty cyanide exposure, establishing large numbers of IV infusion lines by first responders is not feasible. Due to the bandwidth limitations from first responders, this limits the utility of current IV infusion strategies (Meillier and Heller, 2015). To overcome this limitation, a single bolus intramuscular (IM) injection of a cyanide scavenger would enable first responders to treat more patients in a shorter period. Therefore, IM delivery is anticipated to improve survival and limit adverse effects arising from cyanide poisoning (Bhattacharya and Flora, 2015; Brenner *et al.*, 2010; Chan *et al.*, 2015; Kiss *et al.*, 2018; Kovacs *et al.*, 2017, 2016).

Current cyanide countermeasures might have limitations in binding stoichiometry of cyanide. The mechanism of action for hydroxocobalamin involves the direct binding of cyanide in a 1:1 stoichiometric ratio at the metal center (Hamel, 2011). In addition, dicobalt edetate has been of interest for its ability to bind cyanide at up to a 1:2 stoichiometric ratio (Evans, 1964), however dicobalt edetate is associated with significant toxicities (Hall *et al.*, 2009; Marrs and Thompson, 2016). As reported previously, platinum(II) might be an alternative to cobalt which can bind 4 mole equivalents of cyanide (Nath *et al.*, 2017). When administered by IM injection, platinum(II) complexes were efficacious in a mouse cyanide inhalation model at doses as low as 270 µmol/m^2^ of platinum (Behymer *et al.*, 2022). These previous studies have demonstrated that platinum(II)-based complexes could offer an IM available agent with high efficacy by increasing binding ratios between metal and cyanide (Behymer *et al.*, 2022).

In this study, the formulation stabilities of 4 cyanide scavenging Pt(II) complexes **1–4** (Figure 1) were evaluated. The ligands were selected to enhance the reactivity of the metal center by including a thioether group. Each complex was prepared in aqueous media to select for high solubilities and hydrolytic stability. During the original discovery efforts for these bidentate (S,N)-ligand Pt(II) complexes, formation of several pH-dependent mixed isomers were observed, consistent with prior structural assignments (Norman *et al.*, 1992; Summa *et al.*, 2006). However, different isomers of bidentate chelates could have altered cyanide binding kinetics (Appleton *et al.*, 1988). As it is related to formulation stability, the effects of formulation pH may change *in vivo* efficacy that warranted further evaluation.

**Figure 1.**
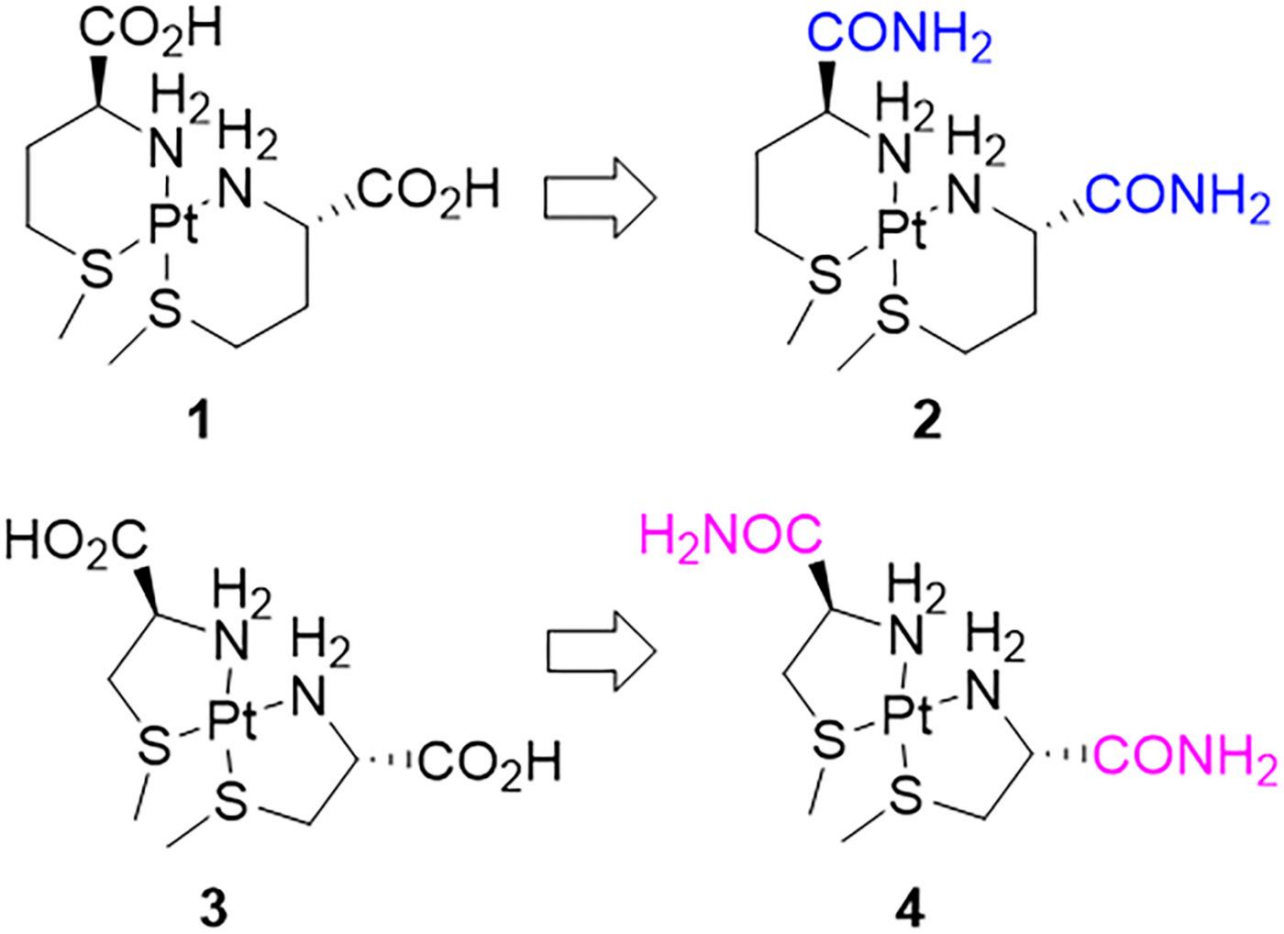
Platinum complexes described in this work. All complexes are representative structures drawn in the *cis* configuration for illustration purposes. Six membered bidentate complexes **1** b*is*-(l**-**methionine (S,N)platinum(II) dichloride and **2** b*is*-(l**-**methionine amide (S,N)platinum(II) dichloride; complex **2** is methionine with carboxamide; 5-membered bidentate complexes **3** bis-(L-S-methylcysteine)-(S,N)platinum(II) dichloride and **4** bis-(L-S-methylcysteine amide)-(S,N)platinum(II) dichloride.

The existing risk of Pt(II)-based therapies to induce adverse side effects can create a dose-limiting toxicity, especially when additional mitigation strategies are not utilized. For instance, the incidence of acute kidney injury (AKI) resulting from the chemotherapeutic agent cisplatin alone is high, occurring in about 30%–40% of patients (Volarevic *et al.*, 2019). Nephrotoxicity from cisplatin therapy in humans is frequently observed at high doses of 200 mg/m^2^ over 5 days and higher, where the dose adjusted for platinum is 664 µmol/m^2^ (Daugaard and Abildgaard, 1989). The objective here is to develop formulations of complexes **1–4** that retain suitable efficacy while significantly reducing the risk for AKI. Our previous results with the novel Pt(II) cyanide scavengers indicated an allometric scaled single dose based on Pt(II) to be 261 µmol/m^2^, well below the therapeutic human dose of cisplatin. Here we hypothesize that the balance of toxicity and efficacy will be reflected in the kinetics of cyanide addition to the Pt(II) complexes. These studies focused on developing formulations of the Pt(II) complexes **1–4** to confirm that *in vitro* reactivity with cyanide can be confirmed using *in vivo* cyanide animal models to ensure efficacy. In addition, the formulations were used to determine the pharmacokinetics upon IM administration and to assess the risk of toxicity. Taken together, the studies here present a strategy for the optimization of a new class of Pt(II) based cyanide countermeasures.

## Materials and methods

Sodium tetrachloroplatinate monohydrate (Na_2_PtCl_4_.H_2_O; Pt(II)) was purchased from Thermo Scientific (Waltham, Massachusetts). Potassium tetrachloroplatinate (K_2_PtCl_4_; Pt(II)) was purchased from Strem Chemicals (Newburyport, Massachusetts). All other chemicals and solvents were purchased from Sigma-Aldrich (Burlington, Massachusetts) or Ambeed (Arlington Heights, Illinois) and used as received. Sodium tetracyanoplatinate anhydrous (Na_2_Pt(CN)_4_) was purchased from Alfa Aesar. Complexes **1–3** with +2NaCl were prepared from K_2_PtCl_4_ as described in Behymer *et al.* (2022).

###  

#### Complex 1 (no NaCl)

A mixture of K_2_PtCl_4_ (208 mg, 0.50 mmol) and l-methionine (75 mg, 0.50 mmol) and Milli-Q water (2.0 ml) was sonicated. The mixture quickly became a dark red-brown colored homogenous solution, and then began to generate precipitates and to change color to light brown, which faded over time. The mixture was sonicated to dissolve all chunks of l-methionine, which immediately generated precipitates, and stirring was continued at ambient temperature in the dark overnight. The solids were isolated from the reaction mixture and washed with Milli-Q water (0.5 ml × 3) to remove KCl, then with isopropyl alcohol (0.5 ml × 2 times) followed by diethyl ether (0.5 ml × 2) and dried *in vacuo* to afford mono(l-methionine)platinum dichloride (MetPtCl_2_,133 mg). A mixture of MetPtCl_2_ (1121 mg), l-methionine (411 mg, 1.02 eq.), and Milli-Q water (2.7 ml) was sonicated to make it homogenous solution, kept at ambient temperature in dark overnight, and lyophilized to isolate complex **1** (1526 mg) NaCl-free batch. Platinum content for this product was approximately 38% w/w Pt (Supplementary Table 1).

#### Complex 2 (no NaCl)

A mixture of K_2_PtCl_4_ (415 mg, 1.0 mmol), l-methionine carboxamide hydrochloride (185 mg, 1.0 mmol), and Milli-Q water (1.8 ml) was sonicated until all solids dissolved. The mixture was kept at the ambient temperature for 5 days in the dark. White solid precipitates were generated, which was isolated by centrifuging the mixture. The solid was resuspended with ice-chilled Milli-Q water (1.0 ml), centrifuged, and supernatant was carefully removed. This water-washing procedure was repeated twice more to remove KCl. The solid was rinsed with isopropyl alcohol (1.0 ml × 2) followed by diethyl ether (1.0 ml × 2), dried to afford mono(l-methionine-carboxamide)platinum dichloride (MetNH_2_)PtCl_2_, 305 mg). A mixture of (MetNH_2_)PtCl_2_ (305 mg), l-methionine carboxamide hydrochloride (139 mg, 1.02 eq.), and Milli-Q water (1.5 ml) was sonicated to generate a homogenous solution, kept at ambient temperature in dark overnight, and lyophilized to isolate complex **2** (452 mg) as a NaCl-free batch. Platinum content for this product was approximately 33% w/w Pt (Supplementary Table 2).

#### Complex 3

See Supplementary Data and Behymer *et al.* (2022).

#### Complex 4 (+2NaCl)

A solution of *N-tert-*butoxycarbonyl-*S*-methylcysteine (2.49 g) in anhydrous tetrahydrofuran (26 ml) chilled by ice bath, and then carbonyldiimidazole (3.78 g) was added stepwise. The mixture was stirred in an ice bath for 4 h, before addition of 28% ammonia solution (6.5 ml) dropwise, followed by stirring an additional 3 h, and storage at 4°C overnight. The mixture was briefly concentrated by rotary evaporation and extracted with ethyl acetate (100 ml). The extract was washed with 1 M HCl (25 ml × 2) to remove imidazole. The aqueous phase was re-extracted with ethyl acetate (20 ml × 1) and the combined extracts were concentrated by rotary evaporation to generate a solid crude residue that was washed with hexanes/ethyl acetate = 2/1 mixture, filtrated, washed with hexanes/ethyl acetate = 2/1 mixture, and dried in open air to afford *N-tert-*butoxycarbonyl-*S*-methylcysteine carboxamide (Boc-SMeCysNH_2_, 2.49 g). The Boc-SMeCysNH_2_ (2.10 g) was dissolved in 1,4-dioxane (9.0 ml) with warming and sonication, before addition of 4 M hydrogen chloride in 1,4-dioxane (9.0 ml, 4.0 eq.). The mixture was kept at the ambient temperature overnight. At this time, the reaction was not complete and more 4 M hydrogen chloride in 1,4-dioxane (4.5 ml, 2.0 eq.) was added. After 4 h, addition of hexanes (23 ml) promoted precipitation of the product that settled to the bottom of the vessel. The supernatant was removed by decantation and the solid was rinsed with diethyl ether (10 ml × 3), with careful decantation each time, and dried *in vacuo* to afford L-*S*-methylcysteine carboxamide hydrochloride (SMeCysNH_2_.HCl, 1.53 g). For the isolated ligand, ^1^H NMR spectrum was acquired to verify the identity (Supplementary Figure 1). Complex **4** (+ 2NaCl) was prepared in an analogous manner for producing complex **1** (+ 2NaCl) described earlier, from Na_2_PtCl_4_.H_2_O (401 mg, 1.00 mmol), SMeCysNH_2_.HCl (345 mg, 2.05 mol eq), and Milli-Q water (5.0 ml), afforded 631 mg. ESI-MS (positive ion, mobile phase: methanol and water), calculated for [C_8_H_22_N_4_O_2_PtS_2_]^2+^: 231.53322; found: 231.53354 *m*/*z*.

#### Osmolality of formulations

Osmolality of each sample was recorded using a VAPRO Vapor Pressure Osmometer Model 5600 (Wescor) with a sample volume of 10 µl.

#### UV-Vis kinetics and platinum content

Rate constants for the platinum spectra were calculated assuming first-order kinetics. In equation 1, *A* is the absorbance. The rate of change (*k*_1_) can be obtained as the slope when $\text{ln}\left(A_{\infty }-A_{t}\right)$ plotted against time (*t*) (Tseng *et al.*, 2010). Each assay was monitored for at least 10 min or until the sample appeared to reach equilibrium. By rearranging equation 1, the half-life can be assessed (equation 2). The observed rate constants and the respective observed half-lives are reported after following the reaction to a minimum of 1 half-life. Platinum content was obtained for each formulation prior to being administered to animals. Each preparation was close to the theoretical amount (eg ±5% w/w). A small reduction in platinum content is possibly attributed to trace excess free ligand and water content of the solid.

(1) $$\ln\left(A_{\infty }-A_{t}\right)=\ln\left(A_{\infty }-A_{t}\right)-k_{1}t$$

(2) $$t_{\frac{1}{2}}=\frac{\text{ln}\left(2\right)}{k_{\text{obs}}}$$

#### HPLC stability assay

An Agilent 1100 equipped with a Diode Array Detector to scan wavelengths 200–300 nm and a Restek Ultra IBD column 2.1 × 50 mm a mixed mode reversed phase/hydrophilic interaction column was used to detect and separate formulation mixtures of the platinum complexes. The column was operated in reverse phase mode with 5 µl injections at 0.3 ml/min. Baseline conditions were 65% purified water with 2 mM ammonium formate and 35% v/v acetonitrile. Elution phase was carried out by a pH 3.7 solution with 2 mM ammonium formate and 0.5% v/v formic acid, gradient to induce ion exchange. Peak identity of Pt(CN)_4_^2−^ was quantified using the purchased standard with detection at 260 nm and identity was confirmed using the absorption spectra for each peak from 200 to 300 nm. Stability samples were stored at room temperature for the duration of the 14-day study. HPLC sample preparation was performed by diluting a 10 mM platinum stock to 350 µM, reacting the platinum by adding KCN to a final concentration of 1.4 mM (1:4 Pt to KCN) for 10 min at ambient temperature. Reaction solutions were 12.5 mM sodium phosphate pH 7.3 buffers. Sample preparations were staggered so each HPLC injection was made at 10 min. The 10-min reaction step was repeated in triplicate for each time point.

#### HPLC Speciation assay

An Agilent 1100 equipped with a UV detector (λ = 220 nm) and Agilent Zorbax Eclipse XD8 C18 column was used with 50 µl injections. Mobile phase consisted of 88% aqueous:12% acetonitrile. The aqueous component consisted of 25 mM sodium phosphate pH 5.5 with 12.5 mM heptanesulfonic acid as an ion-pairing agent. The method was performed using an isocratic run for 35 min at 1 ml/min.

#### Blood-brain barrier permeability model

Permeability was assessed across a novel blood-brain barrier (BBB) triculture model as described previously (Lubin and Knipp, 2021). Briefly, we tested the permeability rates in the Apical (A; blood facing) to Basolateral (B; neuronal side) direction and in the B to A direction to determine the relative brain parenchymal exposure and the efflux ratio (*P*_app,B→A_/*P*_app,A→B_) for **1** (Met_2_PtCl_2_ (+2NaCl)) and **2** (Met-NH_2_)_2_PtCl_2_ (+2NaCl)). For each permeability coefficient determination, 100 µM of each platinum complex was dissolved in Ca^2+^ and Mn^2+^ containing HBSS. The osmolalities of the sample solutions were between 240 and 260 mmol/kg. Permeation rates were determined by loading complex on the apical side for A-B permeability and basolateral for B-A permeability. The receiver chamber had 100 µl sample aliquots taken at 0, 15, 30, 45, 60, 120 and 180 min and analyzed by HPLC. Analysis was carried out at 220 nm using an Agilent Zorbax Eclipse XD8 C18 column.

#### NMR analysis

^195^Pt, ^35^Cl, and ^23^Na spectra were acquired using a Bruker DRX 500 MHz spectrometer equipped with a BBFO probe operating at room temperature. ^1^H and ^13^C spectra were acquired using the same spectrometer or a Bruker Avance 800 MHz spectrometer equipped with a cryo-TCI probe at 25°C. The water-soluble Pt(II) complexes were prepared at 20–100 mM in an aqueous buffer containing 5%–10% D_2_O. Exponential line-broadening window functions (50 Hz for ^195^Pt; 3 Hz for 13C, 0.5 Hz for ^1^H, 1 Hz for ^35^Cl, and 10 Hz for ^23^Na) were applied to FIDs prior to Fourier transformation, manual phasing, and automatic baseline or manual corrections. Cl and Na concentrations were determined by comparison with standard samples of 23.2 or 193 mM NaCl solutions.

#### Sprague Dawley toxicity model

Sprague Dawley rats (Envigo and Inotiv) with weight ranges of 225–250 g were used for this study performed at the Purdue Translational Pharmacology and Clinical Veterinary Pathology Laboratories. Platinum and vehicle were administered by IP injection in ≤5.28 ml/kg at doses listed in Table 2. Blood samples were drawn 1- and 5-days post-injection and processed for Comprehensive Metabolite Panel and Complete Blood Count Panel. Each sample group consisted of 3 males and 3 females unless otherwise specified in the results. At the end of the study, the animals were euthanized following the PHS Policy on the Human Care and Use of Animals, Guide for the Use and Care of Laboratory Animals. All methods were carried out in accordance with the regulations and guidelines of the Animal Welfare Act and the American Association for Accreditation of Laboratory Animal Care. The IACUC committee at Purdue University approved all experimental protocols (1405001069).

**Table 2.** Sprague Dawley formulation details for evaluating toxicity of complexes **1–4.**

Complex	**Dose** $\frac{µ\mathrm{mole}\;\mathrm{Pt}}{m^{2}}$	**Dose** $\frac{\text{mg Pt}}{m^{2}}$	Mouse equivalent dose	pH	Osmolality (mmol/kg)
**1α**	264	51	1×	6.5	1300
**1α**	654	127.8	2.5×	6.5	1060
**1α**	870	169.8	3.3×	6.5	1100
**1α**	1308	255	5×	6.5	1250
**1α**	1308	255	5×	6.5	487
**2α**	1308	255	5×	4.3	600
**2α**	1308	255	5×	6.5	607
**3α**	1338	255	5×	6.6	
**4α**	1308	255	5×	4.3	625

*Note:* Complexes prepared as +2NaCl form (α) or no NaCl form (β).

Complexes **1** and **3** were formulated in phosphate buffer. The final pH of the formulation at the time of injection was 6.5–7.0 using sodium hydroxide for pH adjustment. Complexes **2** and **4** were prepared in sodium acetate buffer (Ca^2+^ and Mg^2+^-free) with pH adjustments using sodium hydroxide to a target pH of 4.2. Final adjustments were made using Milli-Q water to reduce the osmolality of the solution.

#### Sprague Dawley toxicity statistical analysis and software

Analysis was carried out using GraphPad Prism version 10.0.02. An ordinary 2-way ANOVA with a Šidák multiple comparisons test was performed to compare the treated group with the vehicle group means. Data shown is an average ± standard deviation. * = *p* < .05, ** = *p* < .01.

#### Pharmacokinetics protocol

Sprague Dawley rats (Envigo and Inotiv) with weight ranges of 225–250 g were used for this study performed at the Purdue Translational Pharmacology and Clinical Veterinary Pathology Laboratories Rats were injected with either the formulations of complex **1** or **2**, injection volumes of 0.44 ml/kg with a dose of 270 µmol/m^2^. At each interval, 250 µl of blood was sampled and replaced with phosphate-buffered saline using Culex stress-free autosampler units. Samples were collected at 0, 5, 10, 20, 30, 45 m, 2, 4, 8, 12, 24, and 48 h, post administration. At the time of sample collections, blood samples were immediately centrifuged, and the resultant supernatant plasma and blood cell pellet samples were stored in separate vials for each time point at −80°C. Each sample group consisted of 3 males and 3 females unless otherwise specified in the results.

#### ICP-MS digestion

In 15 ml polypropylene centrifuge tubes, 90 µl of rat plasma samples were mixed with 800 µl of 30% v/v hydrogen peroxide (Marcon Fine Chemicals). Subsequently, 800 µl of Aristar ultrapure hydrochloric acid and 400 µl Aristar ultrapure nitric acid were added. The addition of reagents to blood samples resulted in vigorous bubbling. The solutions were loosely capped and incubated at room temperature until bubbling ceased. The sample solutions were then heated at 60°C for 12 h. Each sample was diluted with Milli-Q water to 10 ml and analyzed by ICP-MS.

#### Quantification of platinum by ICP-MS for pharmacokinetics

A ThermoFisher Element II inductively coupled plasma mass spectrometer was used for all Pt(II) analyses. Platinum samples are quantified using tetracyanoplatinate as a standard. Samples were introduced into the mass spectrometer using an Arridus peristaltic pump. Samples were analyzed with take up times of 90 s and sample collection times of 90 s each. Platinum-195 peak intensities were averaged over the sample collection time. These averaged values were converted to nanograms per milliliter using standard curves generated with aqueous solutions of sodium tetrachloroplatinate, prepared by acid digestion similarly to samples.

#### Pharmacokinetic statistical analysis and software

Pharmacokinetic parameters were obtained using a noncompartmental analysis in MatLAB R2023a using the SimBiology Model Analyzer application. Statistics were carried out using a 2-sample *t* test assuming equal variances while comparing the means of complex **1β** and **2β** data. *p* values that are reported were obtained using the 2-tail distribution.

#### Zebrafish cyanide assay

Zebrafish larvae (6 days post fertilization) were loaded (5 per well) into a 96-well plate containing HEPES-buffered Tübingen E3 medium. Preformulated compounds were added to the wells containing zebrafish at an 8-point dose response curve (1–125 µM), immediately followed by the addition of a lethal dose of cyanide (50 µM). The plates were sealed with adhesive microplate sealing films and incubated at 28°C for 3 h. Heart rate and response to touch was used to assess viability (Behymer *et al.*, 2022). The dose that rescued 100% of the larvae in the well (EC_100_) was reported. In a second assay to assess the toxicity of compounds, zebrafish larvae were treated in the same 8-point dose response curve, in the absence of cyanide. Animals were assessed at 3 and 24 h post treatment. Reported survival results are measured 3 h after cyanide exposure. Viability was defined as the presence of a heartbeat. The dose of the drug that induced 100% death (LD_100_) in zebrafish larvae was reported.

#### Lethal mouse cyanide model

C57BL/6 mice were exposed to 587 ppm HCN gas for a total of 40 min as described previously (Behymer *et al.*, 2022; Chan *et al.*, 2015, 2011). After 15 min of HCN exposure, the mice were removed briefly from the exposure chamber and injected intramuscularly with either saline (control animals) or the indicated platinum complex. The mice were placed back in the chamber and exposed to HCN for an additional 25 min. Control animals all died between 29 and 32 min of total exposure time. Animals that survived the HCN exposure were observed continuously for 1 h and then euthanized. Animals that survive for at least 1 h post HCN exposure can be considered true survivors because they will remain alive and well for as long as 1 month post exposure. These studies were approved by the UCSD Institutional Animal Care and Use Committee, Protocol no. S10140.

## Results

### Platinum complexes

Complexes **1–3** (Figure 1) were selected for comparative evaluation of formulation stability with suitable efficacy and safety for advanced large animal testing. The complexes **1** and **2** were previously shown to be reactive with cyanide but differences in efficacies were observed in the lethal cyanide exposure models for zebrafish and mouse (Behymer *et al.*, 2022). In fact, the *bis*-(l**-**methionine amide) (S,N)platinum(II) dichloride **2** demonstrated a reduction in the cyanide reactivity at a pH near 7 (Behymer *et al.*, 2022). Despite the chemical similarity, **1** had consistently higher rates of cyanide scavenging kinetics in comparison to **2** across all assays at neutral pH. These observations motivated a more detailed investigation of this pH dependence on reactivity and the potential for impact on in vivo efficacy.

Platinum complexes are also reported to have greater stability with 5-membered ring systems which might resist the pH-dependent changes (Lawrance, 2013). The enhancement of conformational stability, however, might slow substitution reaction kinetics with cyanide. To investigate if a 5-member ring structure was less reactive, studies with the bis-(S-methyl-L-cysteine) (S,N)platinum(II) dichloride **3** demonstrated enhanced rates of cyanide reactivity in our previous report and similar *in vivo* efficacy to **1** within the resolution of the experimental methods (Behymer *et al.*, 2022). Therefore, the potential role of a carboxamide containing ligand in the 5-membered ring system motivated the preparation of the bis-(S-methyl-L-cysteine amide) (S,N)platinum(II) dichloride **4** to serve as a complimentary test case for the effects of a carboxamide group. Characteristic signals of a free ligand in Supplementary Figure 1 are absent in Supplementary Figure 2, suggesting the ligand is interacting with platinum through a amine and thioether coordination to platinum. The platinum spectrum in Supplementary Figure 3 is very similar to that of **3**, which is also in agreement with Norman’s report for **2** (Norman *et al.*, 1992). Multiple isomers of these species also exist due to the creation of chiral C_α_ and thioether interactions with platinum. Those isomers can readily convert, and was observed to rapidly react with cyanide (Behymer *et al.*, 2022). As a result, no further isomer isolation and identification was attempted. The ^1^H and ^195^Pt NMR spectra of **4** in solution support platinum is complexing with the ligand.

The data in Supplementary Figure 4 were used to obtain an apparent rate constant for Pt(CN)_4_^2−^ production from complex **4.** The initial comparison in UV spectra for **4** in water suggests a pH-dependent change (Supplementary Figure 5), as observed for **2**. The observed rate constant was estimated as 0.6 min^−1^ in presence of 40:1 KCN/Pt. Indeed, cyanide reaction rate with **4** was observed to be slower than **1** (Supplementary Figs. 4A and 4B, respectively).

#### Stability and speciation

**Figure 2.**
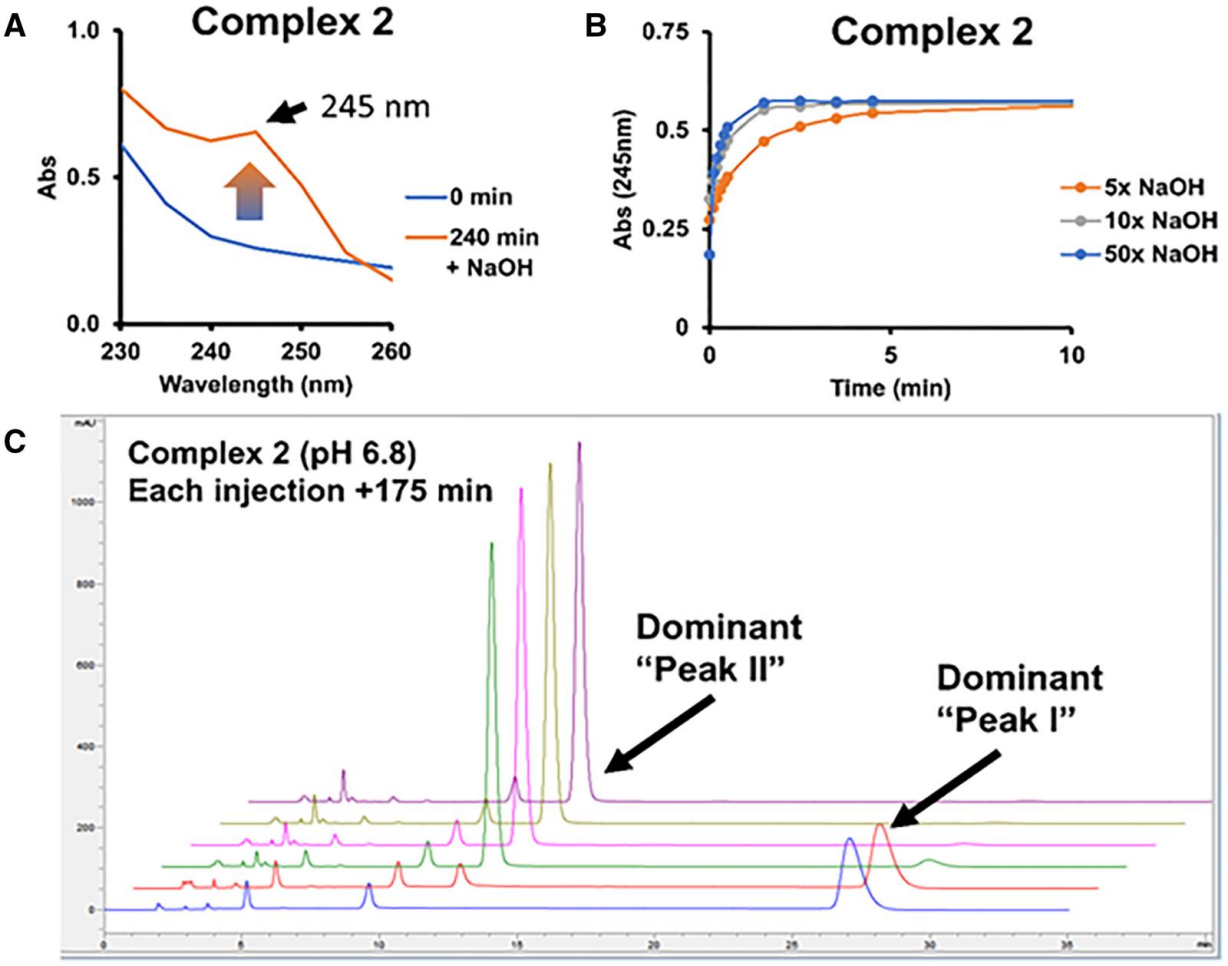
A, Combining **2** with 50 molar equivalents of NaOH and waiting 240 min shows peak development at 245 nm. B, With **2** and varying amounts of NaOH, the signal at 245 nm appeared to increase with increasing NaOH. C, HPLC detection was performed by monitoring the absorbance at 220 nm. When incubated at RT in pH 6.8 phosphate buffer, **2** shows a new form labeled as Peak II by HPLC after 175 min. Blue trace is the control, **2** in purified water and the red trace being the initial mixture at time 0. Molar equivalents are defined relative to the molar level of the Pt(II) complex.

The cyanide scavenging of carboxylate complexes (**1** and **3**) was maintained across all pH conditions (eg, 4–7) at room temperature for up to 14 days. In contrast, the carboxamide complexes (**2** and **4)** had reduced formation of Pt(CN)_4_^2−^ in the formulations at pH > 5 when stored for several days. A potential explanation for the differences is that at elevated pH values a chemical transformation leads to a different species. For instance, complex **2** in purified water revealed spectral changes in presence of sodium hydroxide (Figure 2A). In comparison, **1** and **3** are carboxylate complexes, and they did not demonstrate the same behavior by UV in presence of NaOH. A pH-dependent rate is demonstrated for complex **2** in purified water (Figure 2B), which suggests the potential for a base-catalyzed process. Evaluation by UV at 245 nm for the conversion rate of **2** at 18.8°C, pH 7.3 was found to have a half-life of 2.6 h (Supplementary Figure 6), whereas **4** had an apparent half-life of 3.3 h and 9 min at 19°C and 37°C, respectively (Supplementary Figs. 7A and 7B). HPLC analyses of **2** in pH 6.8 phosphate buffer at room temperature shows gradual changes over 12 h when injected repeatedly possibly due to a speciation event in Figure 2C. Initially there is a dominant form, labeled as Peak I at about 33 min (Dominant Form 1) which disappears after 4 h giving rise to a new Dominant Form 2 labeled as Peak II (Figure 2C).

**Figure 3.**
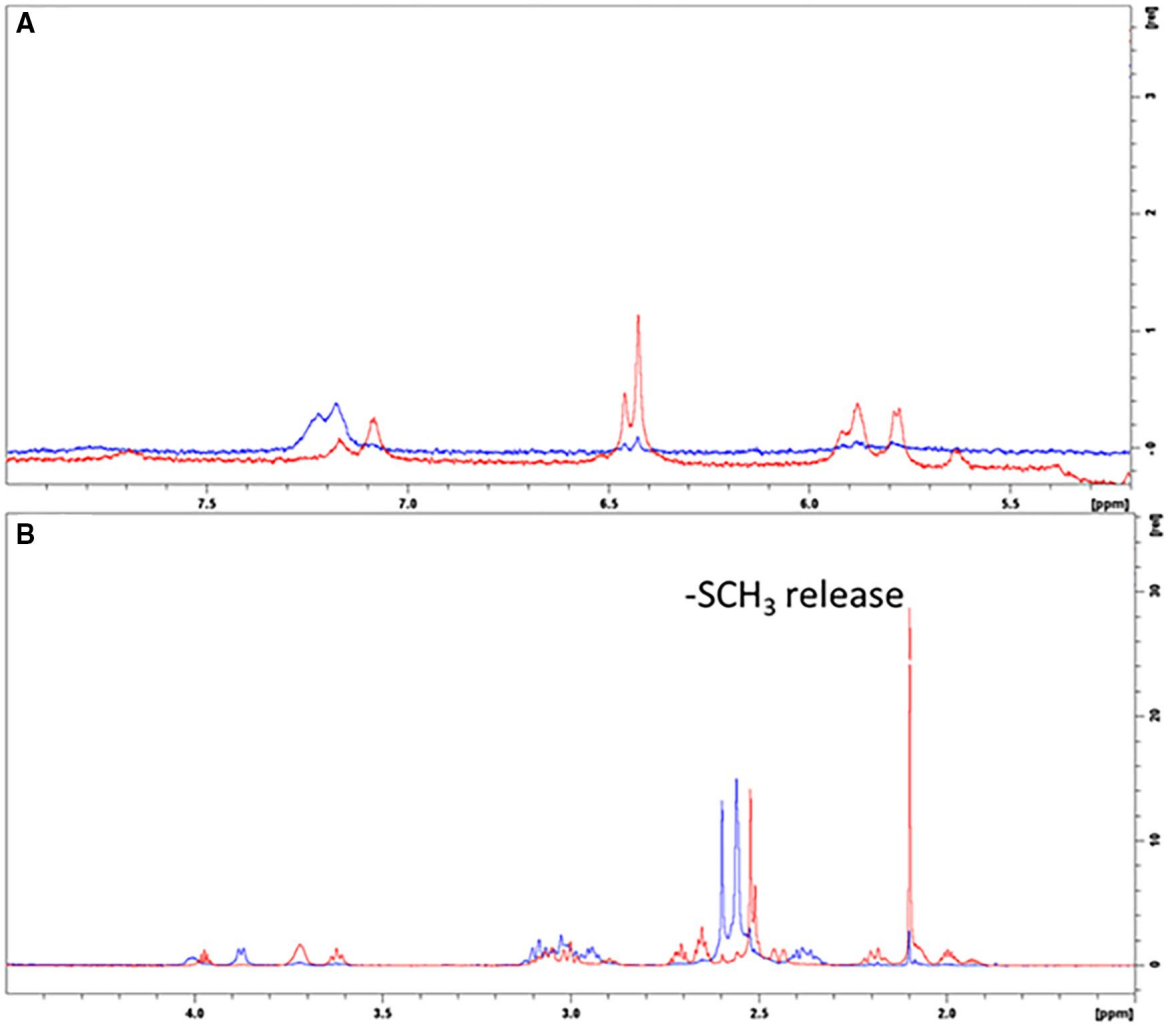
^1^H NMR spectra demonstrates the transformation of complex **2** at pH 7.15 at RT (blue for day 0 and red for day 3). A, Downfield region, for amino and amide NH’s, suggesting their subtle changes over time. B, Upfield regions show changes for the ligand side chain. The loss of thiomethyl binding to Pt is most visible as the signal at 2.1 ppm (consistent with the expected chemical shift for free methionine) grows much more dominant at day 3, whereas the 2 larger signals between 2.6 and 2.55 ppm (day 0) for bound methyl groups shift to 2.5 ppm and become less intense.

The UV and HPLC observations are in general agreement with the NMR data. In Figure 3A, the ^1^H NMR signals at 7.25 and 7.18 ppm become attenuated and shift upfield when comparing day 0 (blue) and 3 (red). In addition, new signals between 5.5 and 6 ppm emerge on day 3. Those changes are generally to the protons of the NH type. On the other hand, Figure 3B demonstrates the concomitant changes for the aliphatic region of the ligand. The singlet signal at 2.1 ppm is assigned as -SMe signal signifying that the functional group was unbound to platinum, as indicated by the significant increase in intensity observed on day 3. The changes in both regions suggest **2** slowly loses interaction between thioether and platinum at pH 7. There is evidence suggesting such a transformation is at least partially pH dependent, as lowering pH (from pH 7) may reverse some of the spectral changes.

**Figure 4.**
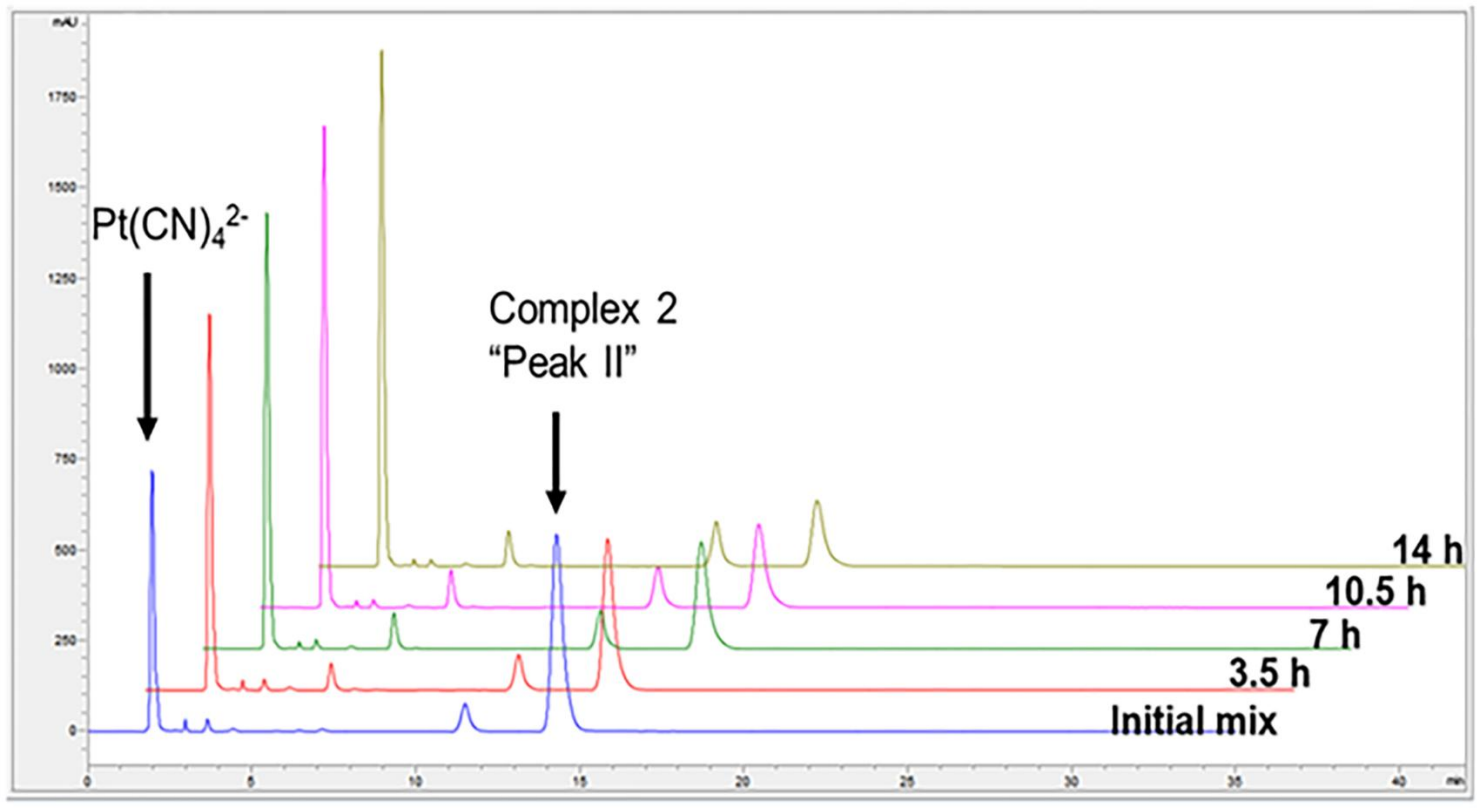
Complex **2** incubated in pH 6.8 phosphate for 3 days at RT. Data shows predominantly “Peak II” at *T* = 0 slowly reacting with 2 mM Complex **2** and 8 mM KCN to produce Pt(CN)_4_^2−^.

The functional stability of the platinum complex formulations is described as their ability to retain reactivity with cyanide during storage in solution. Pt thioether complex **2** which yielded slower reaction rates with cyanide, particularly when stored for several days, was found to be less efficacious in fish and mouse models (Behymer *et al.*, 2022). Upon the addition of 4 mole equivalents of cyanide, form I as shown in Figure 4 immediately disappeared while a slower conversion of Form II occurred over several hours. Pseudo-first-order conditions of complex **2** with cyanide at a 1:40 molar ratio in pH 7 phosphate exhibit a rate of 0.32 min^−1^. Under similar conditions, when **2** is freshly prepared, the observed rate with cyanide was >15 min^−1^ (Behymer *et al.*, 2022). Thus, the rate of cyanide scavenging for **2** stored at a neutral pH is significantly slower than a freshly prepared solution. Under a similar set of conditions, the reaction rate of **4** with cyanide was also diminished (Supplementary Figure 7).

**Figure 5.**
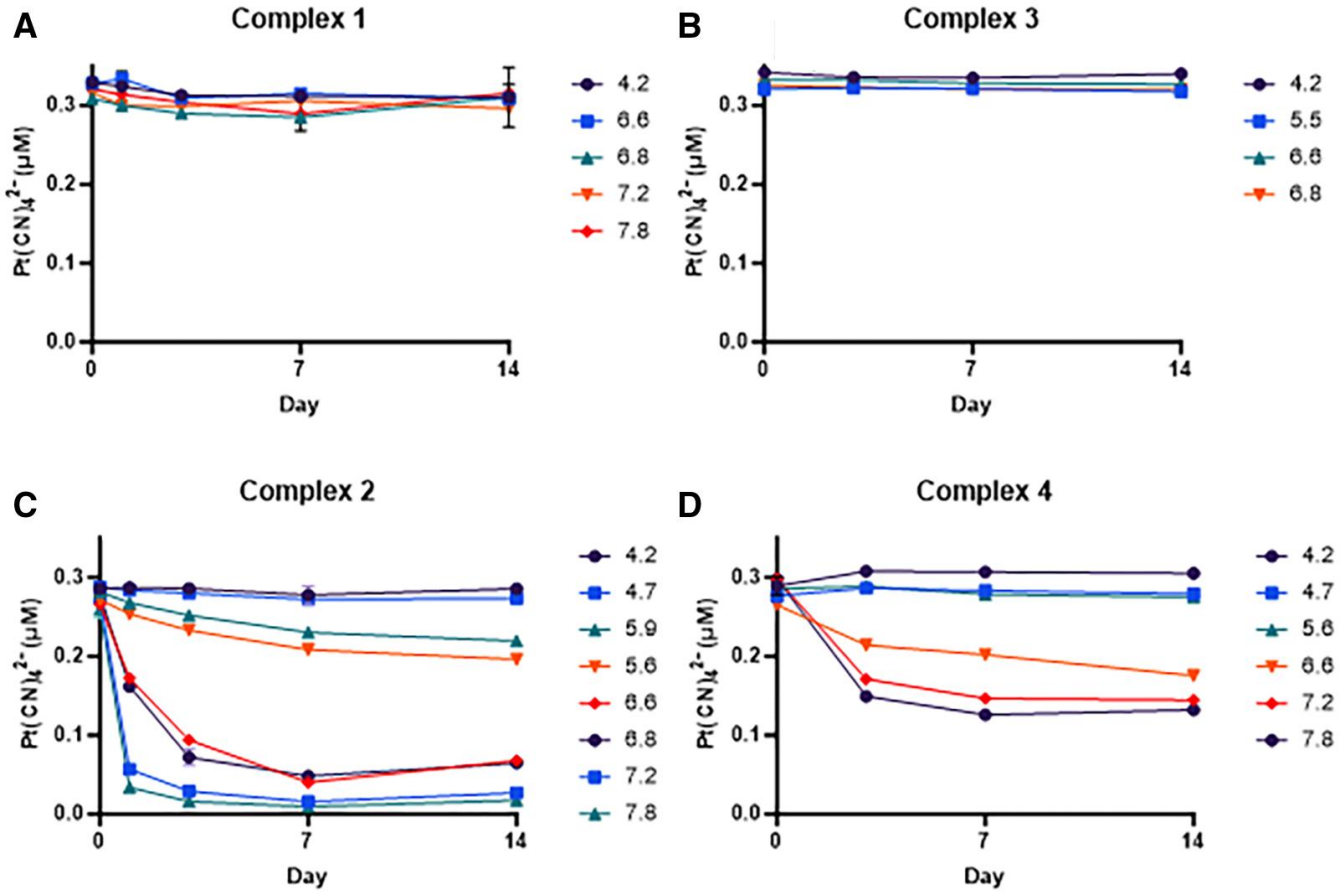
HPLC stability results for complexes **1**–**4** assessed by Pt(CN)_4_^2−^ formation. Each complex was reacted with 4 mole equivalents of KCN for 10 min prior to injection onto the HPLC to quantify Pt(CN)_4_^2−^. Data are presented as mean ± SEM (*n* = 3 replicates/day).

For **2** and **4**, the reactivity loss was observed when the pH of the solution approached neutrality implicating a role for the carboxamide groups distinct from the carboxylate groups in **1** and **3**. To substantiate the apparent differences in reactivity at different pH values, the platinum complexes were stored in a range of buffers for up to 14 days. At different time points, samples of the formulations were reacted with 4 mole equivalents of KCN for 10 min and product (Pt(CN)_4_^2−^) was quantified by HPLC (*n* = 3 for each pH condition) using the purchased reference standard. In all pH conditions, **1** and **3** did not show significant changes in reactivity as evident by Pt(CN)_4_^2−^ production (Figs. 5A and 5B, respectively) over the 14-day period. Monitoring **1** for up to 42 days (Supplementary Figure 8) suggests a potential stability much greater than 14 days. As anticipated, the carboxamide complexes **2** and **4** generated reduced amounts of Pt(CN)_4_^2−^ over the 14 days at pH values ≥ 5 (Figs. 5C and 5D, respectively).

The observed reduction in cyanide-dependent conversion to Pt(CN)_4_^2^ for complexes **2** and **4** upon storage in pH ranges 5–8 in Figures 5C and 5D are consistent with the results observed in Figures 2–4. The pH-dependent changes in forms of **2** and **4** are revealed by monitoring cyanide reactions, where biphasic kinetics are observed leading to significantly slower production of Pt(CN)_4_^2−^ from the second species formed. The data are consistent with the modulation of cyanide scavenging rates of **2** and **4** resulting from a pH-induced isomerization of the complexes to a second form.

Upon reconstitution of the solid preparations for complexes **1** and **2** in water, an acidic solution is formed. Initial acid-base titration of complex **1** (Supplementary Figure 9) reveals an apparent pKa at 2.9. At the equivalence point, approximately 2 moles of NaOH per Pt(II) is consistent with a carboxylate neutralization. However, when the acidic solutions of **1** were stored for an additional 7 days at room temperature before titration with NaOH, a very different profile was observed (Supplementary Figure 9). The extended range of NaOH needed to produce incremental increases in pH was indicative of a complex process. The titration of complex **2** was also complex and did not show obvious equivalence points (Supplementary Figure 10). Furthermore, leaving complex **2** in water for several days did result in a slow acidification of the pH over time after each titration step, which we postulate is due to the complex undergoing a transition to a new form.

#### Efficacy of formulations *in vivo*

**Figure 6.**
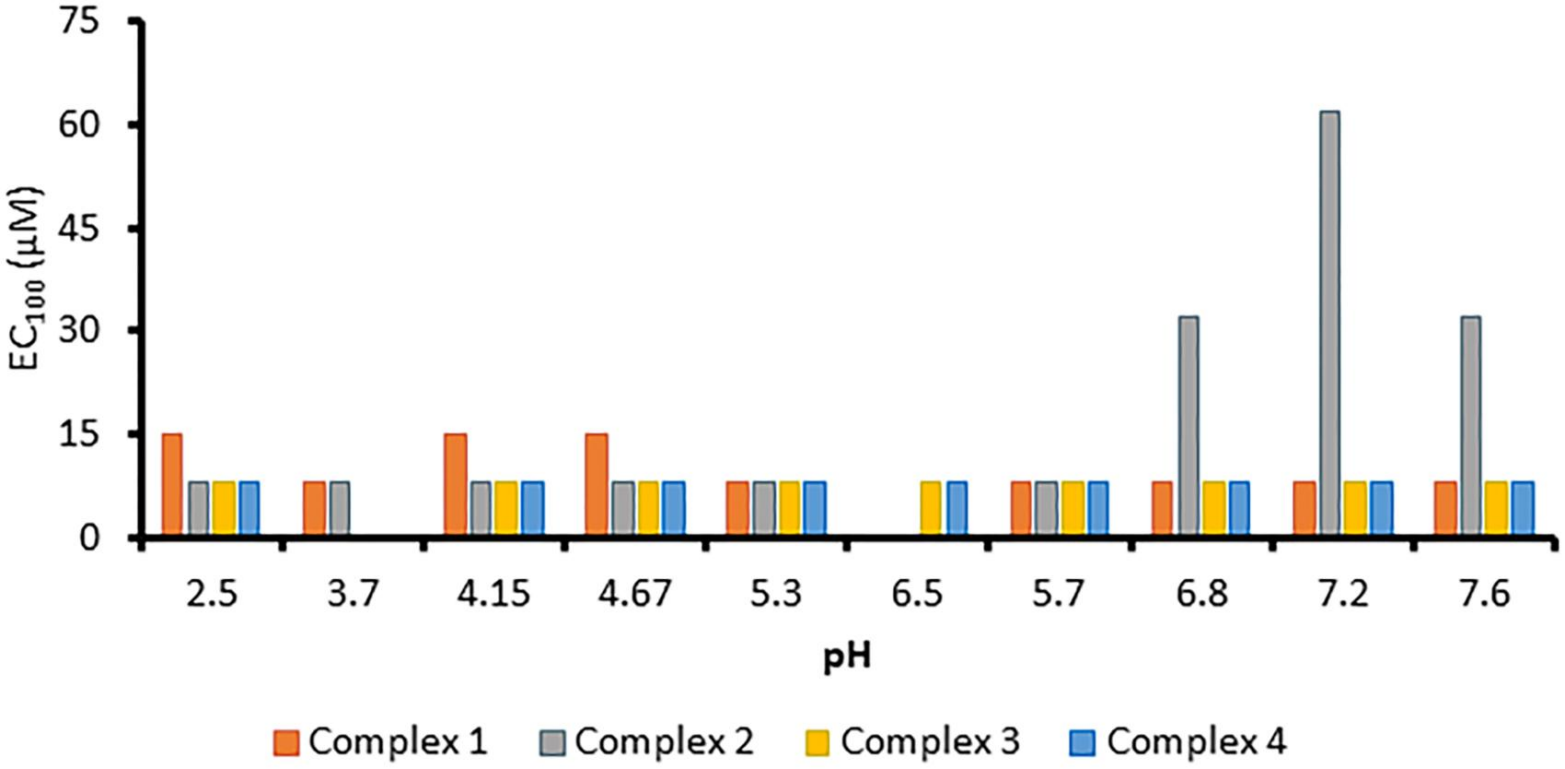
Zebrafish data represents EC_100_ of each complex formulation with aqueous conditions. Each result is the concentration of platinum necessary for 100% (*n* = 5) survival in the presence of 50 µM potassium cyanide, a concentration that results in mortality after 1 h in the control groups. Complexes were prepared 3–7 days prior to use.

Complexes **1**–**4** were first tested in the lethal cyanide challenged zebrafish survival assay to confirm stability for *in vivo* scavenging activity over several days (Nath *et al.*, 2017). Each of the formulations were prepared in buffers from pH 4.3 to 7.6, the details for each formulation are summarized in Supplementary Table 3. Each sample efficacy was tested after 5 days for complexes **1** and **2,** and after 4 days for complexes **3** and **4** to confirm activity. The results in Figure 6 demonstrate that complexes **1**, **3**, and **4** remained efficacious (EC_100_ ≤ 15 µM) across all pH values. Complex **2** had decreased efficacy at pH 6.8 and higher, suggesting that the speciation is having a significant effect on *in vivo* rescue. The zebrafish efficacy results are consistent with the *in vitro* findings for complex **2**, slower cyanide scavenging kinetics occur when **2** is stored near neutral pH values. Toxicity testing was also performed at 3 and 24 h in the zebrafish model, where viability was defined based on the heart rate (Supplementary Table 4). The results demonstrated that at each pH for complexes **1**–**4**, the lethal dose was ≥ 125 µM. Together, these data suggest a lower pH should be considered for the carboxamides stored for several days to achieve an efficacious formulation.

Formulations of complexes **1**–**4** were also tested for efficacy in a previously described lethal cyanide inhalation mouse model via IM injection (Behymer *et al.*, 2022; Chan *et al.*, 2011). A primary motivation for this work was to identify a suitable formulation of complexes **1–4** that retained efficacy by IM injection. A prior study was conducted with complexes **1–3** and prepared as +2NaCl forms (Behymer *et al.*, 2022). Osmolality data with platinum complex concentrations show a linear relationship according to data in Supplementary Figure 11. Analysis of solution content by NMR revealed sodium and chloride is predominately disassociated from the complex as suggested by data in Supplementary Table 5. Therefore, we hypothesized removing NaCl would not have a detrimental impact on IM efficacy. To assess the impact of osmolality on efficacy of IM administration, an alternative procedure to reduce NaCl was investigated. Complexes **1** and **2** were prepared as either +2NaCl (α) or NaCl-free (β) forms and are compared by testing in the mouse lethal cyanide inhalation model using IM administration. Previously, **1α** and **2α** were found to be efficacious for 5/5 and 3/5 mice respectively, when dosed at 261 µmol/m^2^ (Behymer *et al.*, 2022). In Table 1, **1β** had consistent efficacy of 100% survival at the target dose of 216 µmol/m^2^ in the lethal mouse model. Furthermore, the preparations with reduced NaCl content and reduced osmotic pressure also retain efficacy.

**Table 1.** Mouse efficacy and formulation details for platinum complexes **1–4.**

Complex	**Dose** $\frac{µ\mathrm{mole}\;\mathrm{Pt}}{m^{2}}$	**Dose** $\frac{\text{mg Pt}}{m^{2}}$	pH	Survival	Osmolality (mmol/kg)	Reference
**1β**	153	30	6.5	3/4^a^	153	
**1β**	216	42	6.5	4/4	153	
**1α**	261	51	6.5	4/4	565^b^	Behymer *et al.* (2022)
**2β*^a^***	309	60	6	4/4	346	
**2α**	309	60	6.5	1/3	611	
**2β**	168	33	4.2	3/4	210	
**2β**	261	51	4.2	4/4	230	
**2β**	309	60	4.2	4/4	618	
**3α**	291	57	6.5	5/5	608^b^	Behymer *et al.* (2022)
**4α**	309	60	4.2	4/4	625	

*Note:* Formulations were prepared 3–7 days prior to injection.

a Complex 2 was reconstituted and immediately injected intramuscularly.

b Osmolality was estimated using the osmolality concentration curve in Supplementary Data for complex **1**. Complexes prepared as +2NaCl form (α) or no NaCl form (β).

In addition to salt content of the formulation, pH was investigated to optimize the efficacy of formulations. Administering a dose of **2α** at pH 6.5 after aging in solution for approximately 48 h resulted in 1/3 mice survival. A fresh preparation of **2β** at pH 6 was able to provide survival in all 4 mice that were treated at 309 µmol/m^2^. Therefore, to investigate the speciation effect observed in vitro, a formulation at pH 4.2 of **2β** showed efficacy maintained for up to 4 days with 4/4 survival at 261 µmol/m^2^.

We postulated that the 5-membered ring of **4α** might reduce the rate of speciation due to optimized ring strain, improving the stability of the formulation. The results for **4α** revealed total rescue at 309 µmol/m^2^, as indicated in Table 1. The results from the lethal cyanide-treated mouse model demonstrate that low pH can improve the efficacy of intramuscularly delivered platinum complexes **2**(**α** and **β**) and **4α** with the carboxamide ligand by reducing speciation. The work here demonstrates pH significantly impacts the overall efficacy of the carboxamide containing complexes **2** and **4**.

#### Nephrotoxicity of platinum formulations

We utilized the rat model to evaluate dose-dependent changes in blood chemistry and complete blood cell counts to assess the tolerability of a single IP injection of complexes **1α–4α** (Garrett and Korstanje, 2020; Kohl *et al.*, 2020). A link between body surface area and metabolic rate enables allometric scaling to estimate interspecies doses and has been recognized by the FDA (Sharma and McNeill, 2009). For advancement of the complexes, a broader therapeutic safety index (TI) is required, which is defined as the toxic dose relative to efficacious dose to provide a dosing safety window for a therapeutic response. For this work, a dose from mice to rats were allometrically scaled and designed to evaluate if the complexes reach a TI ≥ 5 of the efficacious dose in the lethal cyanide mouse model. The TI is determined by the highest dose used without eliciting changes in clinical safety marker levels shown in Table 2. Animals treated with complex **1α** up to 5 times (1308 µmol/m^2^) the efficacious dose exhibited a significant increase in both blood urea nitrogen (BUN), creatinine (CREA), and phosphate (PHOS) indicating AKI (Supplementary Figure 12). In addition, **1α** was diluted to reduce the formulation of osmotic pressure. No significant difference was observed in the AKI markers between animals treated with high or low osmotic strength **1α** at the highest doses Supplementary Figure 13. Additionally, rats lost approximately 15% body weight when dosed with the highest amounts of **1α** after 5 days consistent with some level of toxic stress caused by a Pt(II) species (Supplementary Figure 14). Complex **3α** also demonstrated significant increases in BUN, creatinine, and phosphate levels after 5 days at approximately 5 times (1338 µmol/m^2^) the efficacious dose.

**Figure 7.**
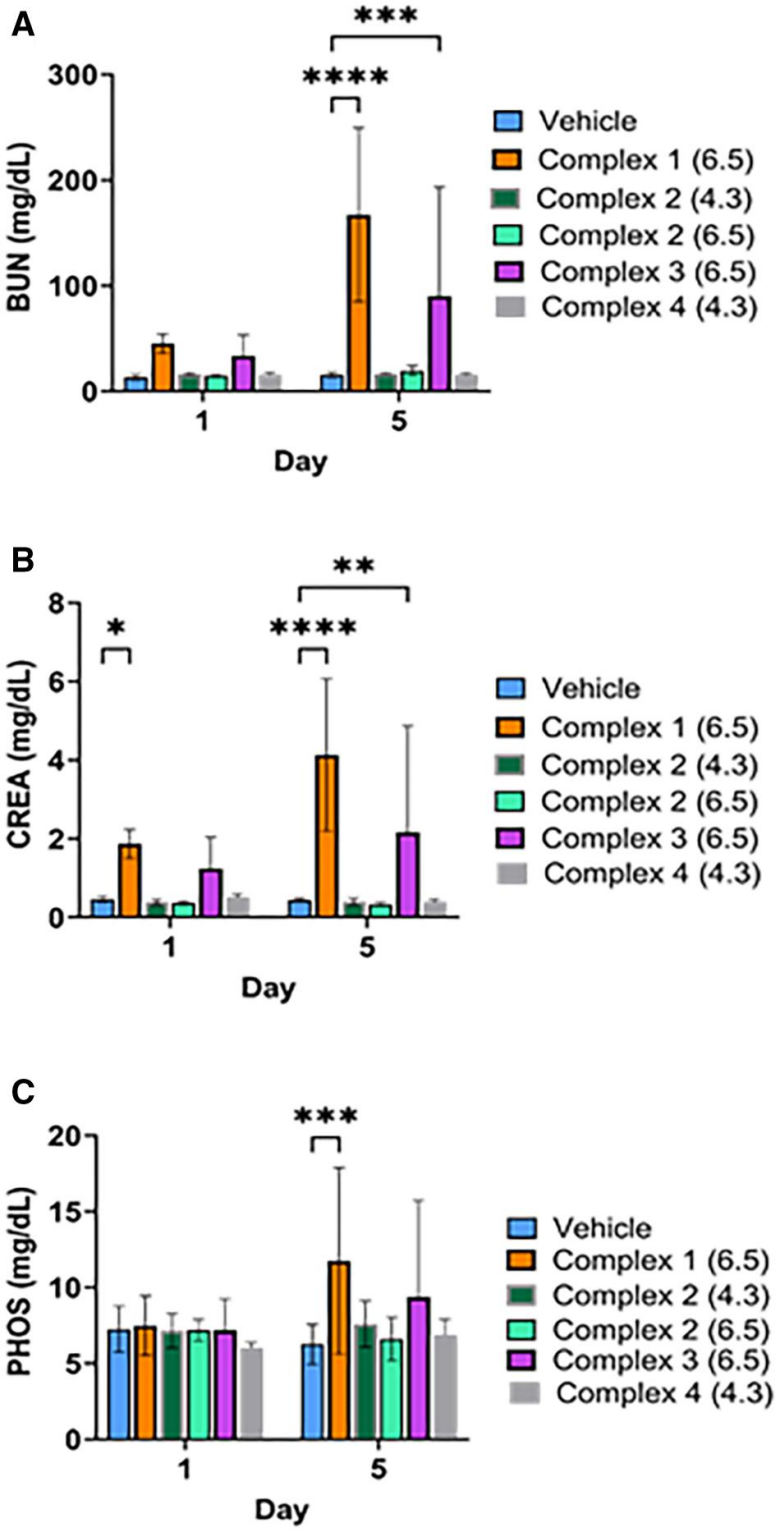
The changes observed in the levels of blood nitrogen urea (BUN), creatinine (CREA), and phosphate (PHOS) in response to the 5× dose of each complex (**1α–4α**). Complex **2α** was assessed at 2 pH values noted within the parentheses. Panels (A–C) show at toxicity as indicated in observed at the highest dose of 1308 µmol/m^2^ (255 mg Pt/m^2^). Data are presented as the mean ± SEM. Asterisks indicate statistical significance at the *p* ≤ .05 (*), *p* ≤ .005 (**), *p* ≤ .005 (***), and *p* ≤ .00005 (****) levels (*n* = 6 rats/treatment 3 male, 3 female).

In sharp contrast to **1** and **3**, the carboxamide containing ligand complexes **2α** and **4α** exhibited minimal effects on the blood chemistry and complete blood counts as shown in Figure 7. Complex **2α** was tested in both pH 4.3 and 6.5 formulations at 5 times (1308 µmol/m^2^) the mouse efficacious dose. No significant differences in renal function biomarkers were observed with either formulation of **2α** when compared with the control group. Rats treated with **4α** at pH 4.3 and 1308 µmol/m^2^ had BUN, CREA, and PHOS levels similar to the control group, and significantly lower than those dosed with **1α** and **3α**. Thus, both **2α** and **4α** have a significantly reduced risk of inducing AKI at 5 times the efficacious dose, and may potentially have an even larger than TI of 5 that is superior to their carboxylate counterparts **1α** and **3α**. Although the study was not designed with adequate power to assess significant gender differences, females do appear to be less prone to increases in the AKI markers from the Pt(II) complexes **1α** (Supplementary Figure 15) and **3α** (data not shown) than males. There are substantial data from animal model studies that highlight gender differences associated with cisplatin-induced toxicities (Marcu, 2022). In addition, to confirm that the Pt(CN)_4_^2−^ scavenging product from the complexes would not pose a higher threat of AKI in patients, we demonstrated that no significant renal toxicity was observed after 5 days at a dose of up to 871 µmole/m^2^ (Supplementary Figure 16). Finally, significant differences were not observed in the complete blood count values between animals treated with complexes and controls (data not shown).

#### Pharmacokinetics of intramuscular formulation

**Figure 8.**
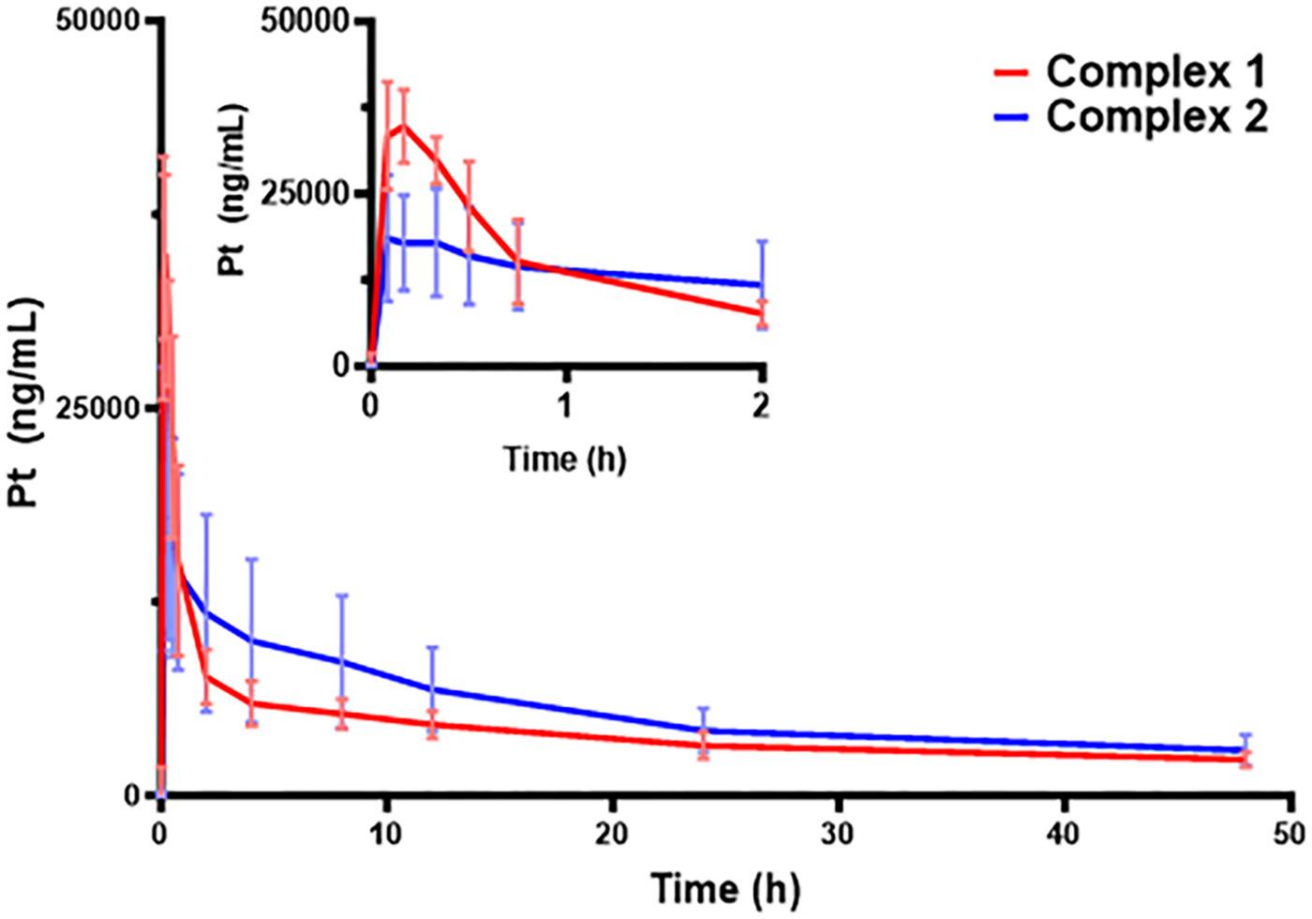
Total platinum in plasma concentration versus time profiles, as measured by ICP-MS, in rats for complex **1β** and **2β**. The expanded earlier concentration versus time profile (inset) in the figure illustrates the apparent differences in distribution early on between the 2 complexes. Data are the mean ± SEM (*n* = 5 rats/treatment 2 male, 3 female).

A proof-of-concept study was performed to assess the similarities and potential differences in the pharmacokinetic parameters between complex **1β** and **2β** in rats. The data obtained will be used to estimate the doses needed upon allometric scaling to achieve efficacy in future studies. The study design involved 2 groups of 6 rats (3 male and 3 female), each when dosed with 270 µmol/m^2^ of either **1β** or **2β**. Technical challenges with the Culex autosampler resulted in a final sample group of 2 males and 3 females for each dose. Each complex was administered IM to mimic the efficacy model and product concept. Plasma levels of total Pt in animals were monitored for 48 h (Figure 8). These data were evaluated for differences in absorption rates, elimination rates, and overall exposure by AUC values (Table 3). Both complexes appear to reach maximal concentrations within 7–9 m after IM administration, which is consistent with a highly diffusive agent that is capable of rapid absorption. However, significant differences between the dose normalized maximal concentration ($\frac{C_{\max}}{\mathrm{dose}}$) in plasma were observed where **1β** is about 2.2 times greater than that for **2β**.

**Table 3.** Pharmacokinetic parameters for **1β** and **2β** obtained from intramuscular injection in Sprague Dawley rats.

Parameter	Complex 1β	Complex 2β	*p* value
*C*_max_/dose (1/l)	16 805 ± 2196	7685 ± 3245	>.005
*T*_max_ (h)	0.150 ± 0.109	0.117 ± 0.0456	.547
MRT (h)	59.2 ± 30.9	39.4 ± 10.5	.213
Elim. *T*_1/2_ (h)	43.9 ± 20.9	27.8 ± 8.48	.148
AUC_0–48_ (g*h/l)	0.203 ± 0.0329	0.271 ± 0.104	.203
AUC_0–∞_ (g*h/l)	0.361 ± 0.0977	0.386 ± 0.128	.736
AUC_0–∞_ Dose (h/l)	169 415 ± 45 866	151 988 ± 50 456	.583
CL (ml/h)	6.21 ± 1.47	7.28 ± 2.70	.463

*Note:* Data are the mean ± SEM (*n* = 5 rats/treatment 2 male, 3 female).

Figure 8 inset highlights a biphasic disposition profile observed for **1β**, which suggests a dominant distribution phase in the first 2 h. In contrast, **2β** appeared to have a steady disposition phase, where the distribution phenomena were not kinetically different from the elimination rate. Despite these possible differences, the AUC and clearance rates revealed a lack of statistically significant differences between the 2 complexes. The extrapolated clearance rates and the terminal elimination half-lives for **1β** and **2β** were also not statistically different. These elimination rates appear faster than those reported for other platinum drug complexes in rats (Wang *et al.*, 2007). Together, these results highlight that **1β** and **2β** have utility as cyanide scavenger agents with rapid absorption by IM administration, but also point to different disposition outcomes despite the relatively small structural differences. Further investigation is needed to identify the underlying reasons for these differences in disposition.

Cyanide can be a potent neurotoxin, thus distribution of **1β** or **2β** into the brain may help to reveal the potential to mitigate neurotoxicity. To evaluate the potential for brain distribution, the permeability of complexes **1β** and **2β** was assessed across an *in vitro* BBB triculture model as shown in Table 4. The permeability coefficients compare favorability with rates determined for established markers having higher *in vivo* brain distribution and permeation across the BBB (Kulczar *et al.*, 2017). The BBB permeation rates suggest that both complexes may be able to scavenge cyanide in the brain parenchyma.

**Table 4.** The apparent permeability (P_app_) of **1α** and **2α** across a direct contact BBB triculture model.

	A-B permeability (×10^−5^ cm/s)	B-A permeability (×10^−5^ cm/s)
Complex **1α**	1.95 ± 0.27	2.15 ± 1.28
Complex **2α**	1.84 ± 0.19	1.81 ± 0.04

Note: Results shown are the mean ± SEM (n = 3).

## Discussion

Ligands such as l-methionine can produce S,N-chelates with platinum, and can exist in solution as multiple isomers (Behymer *et al.*, 2022; Norman *et al.*, 1992). Prior evidence for complex **2α** suggested that the different conditions for cyanide scavenging between assay methods may lead to an explanation for the differing antidotal efficacies (Behymer *et al.*, 2022). As shown in this work, complexes **2α** and **4α** display pH dependences on cyanide reactivity that coincides with forming a new species. The association rate to platinum by amines increases at a higher pH, with losing a proton to encourage Pt–N bond formation (Appleton *et al.*, 1988, 1985; Summa *et al.*, 2006). The observed pH dependence differences in stabilities between carboxylates (**1α** and **3α**) and carboxamide ligands (**2α** and **4α**) support a mechanism of intramolecular isomerization, possibly via amine deprotonation as a critical step to facilitate ring closure. Pt–ligand bond angles in a 5-membered ring should have greater stability than 6-membered structures based on the Pt–S/N bond lengths being shorter (Lawrance, 2013). As indicated by UV and HPLC, the results indicate the rate of new species formation is slower for **4α** than **2α**. In our previous study, **3α** showed slower cyanide scavenging rates than **1α** reflecting higher conformational stability resulting in slower substitution kinetics with cyanide (Behymer *et al.*, 2022). A reduced cyanide-scavenging rate was also observed under the same conditions for **4α**. These observations suggest a potential for the S,N-chelate size to influence isomerization and cyanide scavenging rates, with 5-member ring structures being more stable than 6-member structures bound to Pt(II).

The formulation pH conditions for IM administration were screened to identify optimal conditions to maintain cyanide scavenging for each Pt(II) complex. Our findings revealed that complexes **1α** and **3α** maintain similar kinetic activity towards cyanide across the pH ranges studied. However, complexes **2α** and **4α** have reduced cyanide-scavenging kinetics when formulated above pH 5 after 3 days. Complexes **2** and **4** were stable and maintained reactivity at pH ranges ≤ 5. Optimal formulation pH conditions were determined for all 4 complexes and lead to the rapid stoichiometric formation of Pt(CN)_4_^2−^ exceeding the binding of any known scavenger approved or in development. Reduced osmotic strength from diluting the **1α** and **2α** formulations were determined to have little impact on the nephrotoxicity and reactivity of platinum. Therefore, to alleviate the hypertonic IM injection conditions and reduce osmotic pressure, lower NaCl containing starting material was used to generate **1β** and **2β** and then compared with the high salt preparations **1α** and **2α** (Behymer *et al.*, 2022). For these complexes, osmolality and NMR results suggest nearly complete disassociation of Cl^−^ from platinum starting materials.

Another critical quality for countermeasures in pharmaceutical development is a suitable safety profile. A significant challenge to diagnose cyanide exposure is presented with victims and necessitates developing drugs that elicit minimal adverse toxicities to reduce compounding effects (Seyit *et al.*, 2021). AKI is commonly encountered in patients receiving cisplatin which is dependent on dose, dose frequency, and cumulative dose (Miller *et al.*, 2010). Cumulative nephrotoxic dose of cisplatin in humans may occur at 667 µmol/m^2^ (Daugaard and Abildgaard, 1989). Carboplatin, a cisplatin analog is less nephrotoxic however still shows decreased renal function in patients at cumulative doses ≥ 2154 µmol/m^2^ (Cornelison and Reed, 1993). As such, evaluating the risks of toxicities like AKI associated with platinum complexes is critical. Sprague Dawley rats are well-recognized as a sensitive model for detecting platinum-induced nephrotoxicity (Perše and Večerić-Haler, 2018). At doses 5 times the efficacious dose in mice, **1α** and **3α** resulted in evidence of AKI after 5 days. These results consistent with observations in cisplatin-treated Sprague Dawley rats when co-administered methionine (Basinger *et al.*, 1990; Jones *et al.*, 1991a). Interestingly, carboxamides in complexes **2α** and **4α** significantly reduce the incidence of observed AKI in the rat model. The levels of platinum that were dosed in the rats here far exceed the lethal rat dose of platinum found in cisplatin, suggesting that these complexes afford safety beyond that of the approved chemotherapeutic (Perše and Večerić-Haler, 2018).

Efforts to reduce nephrotoxicity for platinum-based therapeutics and improve safety has been an active area of research (Miller *et al.*, 2010). In fact, l-Methionine and d-Methionine have been used to ameliorate the nephrotoxic effects of cisplatin in rats (Basinger *et al.*, 1990; Jones *et al.*, 1991a; Jones and Basinger, 1989; Lin *et al.*, 2018). This reduction in platinum-induced AKI from cisplatin is hypothesized to be due to reduced reactions with thiol containing amino acids, peptides, and proteins in the cytosol (Stankovic *et al.*, 2020). Thioethers and other strongly binding ligands to platinum have been proposed to function as antioxidants (Stankovic *et al.*, 2020). For instance, sulfhydryl supplied by glutathione or anion sulfur ions (eg, thiolate and WR-2721) have been used to mitigate cisplatin-induced nephrotoxicity (Jones and Basinger, 1989). The most promising antioxidants such as dithiocarbamates and WR-2721 show decreased renal platinum levels (Jones *et al.*, 1991b).

In addition to nephrotoxicity, other platinum-based drug products have known ototoxicity, cardiotoxicity, and peripheral neuropathy risks (Barefoot, 2001; Oun *et al.*, 2018). The ototoxicity of cisplatin has been shown to mitigated with d-methionine in a Phase 2 clinical study (Campbell *et al.*, 2022). As stated by Egorova *et al.* toxicity of a metal ion is not uniform and considerations for oxidation state, ligands, and other physiochemical characteristics are differentiators (Egorova and Ananikov, 2017). For example, renal transporters and tissue accumulation are believed to play a role in platinum nephrotoxicity (Pabla and Dong, 2008). Carboplatin, a cisplatin analog has reduced nephrotoxicity and is proposed to be a result of reduced transporter interaction and protein binding (Małyszko *et al.*, 2017; Martinez *et al.*, 1993). Zebrafish exposed to Pt(CN)_4_^2−^ up to 1000 µM showed no signs of toxicity or gross morphological, far above the efficacious dose for complexes **1–4** (Nath *et al.*, 2017). In addition, the markers for hepatotoxicity in Sprague Dawley rats also suggested minimal toxic side effects for **1–4** and Pt(CN)_4_^2−^. Despite these promising results, further investigations of toxicity need to be initiated before other mechanisms of toxicity can be eliminated.

For the acute cyanide scavenging setting, using rapid-acting platinum rescue agents offers benefits as a single bolus IM dose. Although a correlation between Pt reactivity and toxicity has been discussed previously (Oun *et al.*, 2018), this study reveals that the platinum complexes with carboxamide ligands can slowly convert to less toxic forms to create agents with improved safety. These comparisons of **1α**–**4α** afford insights into the potential balance needed to achieve the safety and efficacy of S,N-chelated Pt(II) complexes with a therapeutic index of 5 or greater.

## Conclusions

The proposed mechanism for a highly efficacious cyanide scavenger is predicated on rapid chelation in a bolus dose with a minimal risk of toxic effects. We have identified 4 complexes based on (S,N)Pt(II) ligation with methionine and S-methylcysteine with the free and amidated carboxylates. All 4 complexes operate in optimal pH ranges to react with a 1:4 stoichiometric formation of Pt(II):CN. The data provide evidence that the free carboxylates of each amino acid yield fast cyanide scavenging Pt(II) complexes **1α** and **3α** but may also increase AKI at higher doses. Alternatively, the carboxamides (**2α** and **4α**) on the bidentate amino acid ligands have reduced scavenging kinetics when formulated at pH > 5. However, an optimized pH formulation of the carboxamide complexes maintained cyanide reactivity while offering a significant reduction in the risks of platinum-induced AKI. These observations have provided criteria essential for the generation of potentially translatable (S,N)Pt(II) cyanide scavengers that will operate within a broader therapeutic window while maintaining a 1:4 stoichiometric binding of Pt:CN.

## Supplementary Material

kfad119_Supplementary_Data

## Data Availability

The data in this manuscript are available in the manuscript and the online Supplemental Material.
