# Supplementary material for: Investigating the replacement of carboxylates with carboxamides to modulate the safety and efficacy of platinum(II) thioether cyanide scavengers
Source: Toxicol Sci. 2023 Nov 11;197(2):197–210. doi: 10.1093/toxsci/kfad119 (PMC10823771; doi:10.1093/toxsci/kfad119)
Supplement: kfad119_Supplementary_Data [file kfad119_supplementary_data.docx]

**Supporting Data**

Preparation of Complexes **1-3** (+2NaCl) was completed using the protocol described in Behymer et al. (Behymer *et al.*, 2022).

**Characterization Methods**

**Mass spectrometry (high resolution)**: Platinum agents were dissolved in 50:50 methanol and water solvent. The sample was infused into a LTQ Orbitrap mass spectrometer using electrospray ionization in either positive or negative mode.

**UV-Vis kinetics:** The rates of change for the platinum spectra are plotted as first order kinetics. Where in Equation (1), the rate of change (k_1_) is obtained by a linear transformation of data:

$$\left( \text{1} \right)\text{ ln}\left( \text{A}_{\text{∞}}\text{-}\text{A}_{\text{t}} \right)\text{=}\ln\left( \text{A}_{\text{∞}}\text{-}\text{A}_{\text{t}} \right)\text{-} \text{k}_{\text{1}}\text{t}\text{.}\text{ }$$

**UV-Vis platinum content:** Preparations of each platinum complex were prepared in MilliQ purified water with ≥ 5:1 cyanide:Pt. Each vial with cyanide and platinum was sealed and reacted for a minimum of 24 hours at room temperature. The platinum as Pt(CN)_4_^2-^ was quantified using the absorption at 255 nm.

**Zebrafish toxicity:** TubigenAB zebrafish embryos (bred in-house) were incubated with each compound at the indicated doses. The heart rate changes observed for each compound and assessed for signs of cardiotoxicity or lethality (Behymer *et al.*, 2022).

**Acid-base titration:** Each complex was prepared in water at approximately 2 mM. Using a Class A burette, 5 mM sodium hydroxide was titrated into the sample solution with a solution pH > 10 as the endpoint.

**NMR:** The dried powder samples were dissolved and analyzed by 195Pt NMR to monitor the deshielding effect on the platinum core and observed as shifts in the ≤ -2800ppm with hexachloroplatinate reference.

**References**

Behymer MM, Mo H, Fujii N, Suresh V, Chan A, Lee J, Nath AK, Saha K, Mahon SB, Brenner M, et al. 2022. Identification of Platinum(II) Sulfide Complexes Suitable as Intramuscular Cyanide Countermeasures. Chem Res Toxicol [Internet]. [accessed 2022 Oct 9]. https://doi.org/10.1021/acs.chemrestox.2c00157

Norman RE, Ranford JD, Sadler PJ. 1992. Studies of platinum(II) methionine complexes: metabolites of cisplatin. Inorg Chem [Internet]. [accessed 2021 May 29] 31(5):877–888. https://doi.org/10.1021/ic00031a033

**Table S1.** Platinum content for complex **1** (no NaCl). Absorption data uses the diluent as a referenced blank. Data shown is the platinum analysis acquired by UV-Vis and potassium cyanide after 24 hours. The results are averages of n=3 replicates with < 0.5% RSD showing good reproducibility for the UV-Vis method.

| **Source** | **Absorbance**  **(255 nm)** | **Pt(CN)_4_^2-^ Cuvette (mM)** | **Pt(CN)_4_^2-^ Stock Calc. (mM)** | **Pt % w/w** |
| --- | --- | --- | --- | --- |
| Na_2_PtCl_4_ precipitation | 0.669 | 0.0698 | 9.7374 | 37.98 |
| Na_2_PtCl_4_ precipitation | 0.669 | 0.0698 | 9.7338 | 37.96 |
| Na_2_PtCl_4_ precipitation | 0.674 | 0.0703 | 9.8139 | 38.27 |

^†^Analysis of percent platinum content for each replicate resulted in an average ± standard deviation of 38.1 ± 0.18.

**Table S2.** Platinum content for two batches of **2** (no NaCl). The data shown is the platinum content analysis acquired by UV-Vis and potassium cyanide. The results shown are the average of 3 replicates with 32% and 33% %w/w Pt for Batch A and batch B respectively. These results show synthesis of **2** (no NaCl) may reliably produce a solid material with consistent platinum content. *Absorption at 255 nm was recorded after background subtraction using the diluent as a reference.

| **Preparation #** | **Source** | **Absorbance** | **Pt(CN)_4_^2-^ Cuvette (mM)** | **Pt(CN)_4_^2-^ Stock Calc. (mM)** | **Pt %w/w** | **Mol Ratio of Pt:Na:Cl** |
| --- | --- | --- | --- | --- | --- | --- |
| 1^†^ | PtCl_2_ | 0.799 | 0.083 | 8.31 | 32.41 | 1:0:2 |
|  | PtCl_2_ | 0.776 | 0.081 | 8.08 | 31.49 |  |
|  | PtCl_2_ | 0.791 | 0.082 | 8.23 | 32.10 |  |
| 2^††^ | PtCl_2_ | 0.822 | 0.086 | 8.56 | 33.38 | 1:0:4 |
|  | PtCl_2_ | 0.811 | 0.084 | 8.44 | 32.92 |  |
|  | PtCl_2_ | 0.813 | 0.085 | 8.47 | 33.01 |  |

^†^Analysis of percent platinum content for each replicate resulted in an average ± standard deviation of 32.0 ± 0.47.

^††^Analysis of percent platinum content for each replicate resulted in an average ± standard deviation of 33.1 ± 0.24.

**Table S3.** Zebrafish formulation details are summarized. Several buffer conditions were prepared for each complex (1-4) at approximately 10x the platinum concentration to ensure stable pH during shipment.

| **Buffer** | **pH of Formulation*** |
| --- | --- |
| Acetate | 3.77 |
| Acetate | 4.2 |
| Citrate | 4.8 |
| Citrate | 6.0 |
| MES | 5.5 |
| Phosphate | 7.0 |
| HEPES | 7.00 |
| Tris | 7.20 |
| Bis-Tris | 6.5 |
| Water | ~2.5 |

**Table S4.** Toxicity in zebrafish is represented as the dose of the drug, in the absence of cyanide that induced 100% death in zebrafish larvae. Animals were assessed at 3 and 24 hours post treatment. Viability was defined as the presence of a heartbeat of the control group; lower heart rates indicate increased cardiotoxicity (n=12 per group).

|  | **Complex 1β** | | **Complex 2 β** | | **Complex 3 β** | | **Complex 4α** | |
| --- | --- | --- | --- | --- | --- | --- | --- | --- |
| **pH** | **3 hr** | **24 hr** | **3 hr** | **24 hr** | **3 hr** | **24 hr** | **3 hr** | **24 hr** |
| **2.5** | >125 | >125 | >125 | >125 | >125 | >125 | >125 | >125 |
| **3.7** | >125 | 125 | >125 | >125 | - | - | - | - |
| **4.15** | >125 | >125 | >125 | >125 | >125 | >125 | >125 | >125 |
| **4.67** | >125 | >125 | >125 | >125 | >125 | >125 | >125 | >125 |
| **5.3** | >125 | >125 | >125 | >125 | >125 | >125 | >125 | >125 |
| **6.5** | - | - | - | - | >125 | >125 | >125 | >125 |
| **5.7** | >125 | 125 | >125 | 125 | >125 | >125 | >125 | >125 |
| **6.8** | >125 | >125 | >125 | >125 | >125 | >125 | >125 | >125 |
| **7.2** | >125 | >125 | >125 | >125 | >125 | >125 | >125 | >125 |
| **7.6** | - | - | - | - | >125 | >125 | >125 | >125 |

*The highest dose tested was 125 µM. The red text denotes the LD_100_ was found. Dashes denote a compound/pH combination was not assessed.

**Table S5.** Ion content for each complex evaluated by NMR. Values were obtained by integrating ^1^H, ^23^Na and ^35^Cl using water as a reference spectra.

| **Complex**** | **Complex (mg/mL)** | **Estimated Concentration* (mM)** | **Free**  **Na^+^ (mM)** | **Free**  **Cl^-^ (mM)** | **Observed Fraction**  $\frac{\text{Cl}^{\text{-}}}{\text{Pt}}$ |
| --- | --- | --- | --- | --- | --- |
| 1 α | 13.2 | 15 | 62 | 90 | 6 |
| 1 β | 2.2 | 3.9 | 0 | 6 | 1.5 |
| 2 α | 4.4 | 4.6 | 14 | 34 | 7.4 |
| 2 β | 20.54 | 33.5 | 6 | 111 | 3.3 |
| 3 α | 23.7 | 25 | 109 | 173 | 6.9 |
| 4 α | 17.36 | 17 | 74 | 162 | 9.5 |
| ^*^Concentrations for complexes are estimated by integrating the signals in ^1^H spectra using water as a reference and subtracting the disassociated ligand.  **Complexes prepared as +2NaCl form (α) or no NaCl form (β). | | | | | |

**Supplemental Figures**

*
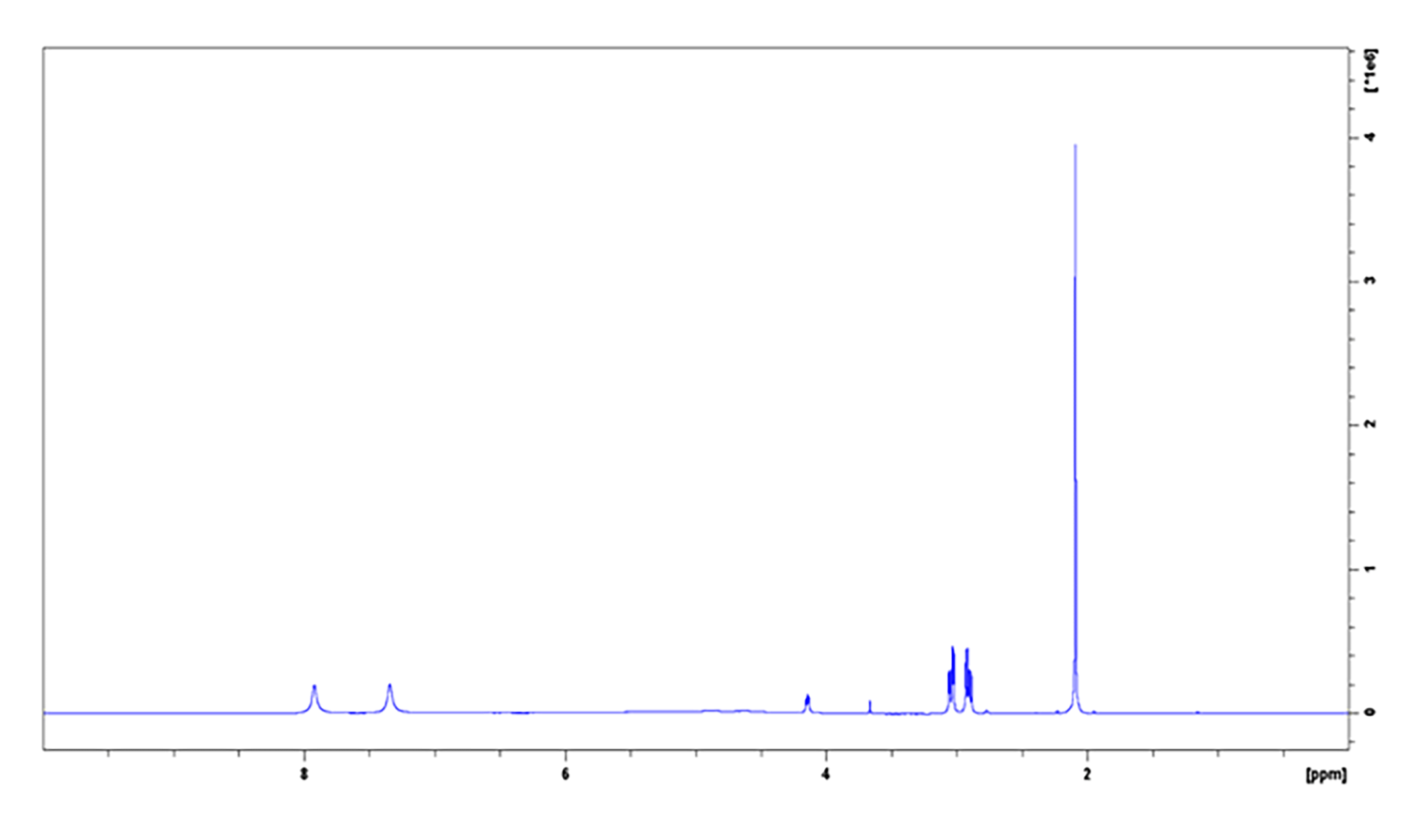
*

**Figure S1.** ^1^H NMR (500 MHz) for a fresh solution of free ligand (SMeCys-NH_2_) in MilliQ purified water with 5% v/v D_2_O at 293 K. S-methyl group is clearly observed as a sharp singlet at 2.1 ppm. While two broad signals at 7.92 and 7.35 ppm are assigned to the amide NH_2_, the amino group is suppressed by presat water suppression due to its fast proton exchange with water. The other signals at 4.15 and 3.04/2.91 ppm are assigned to CH and CH_2_.


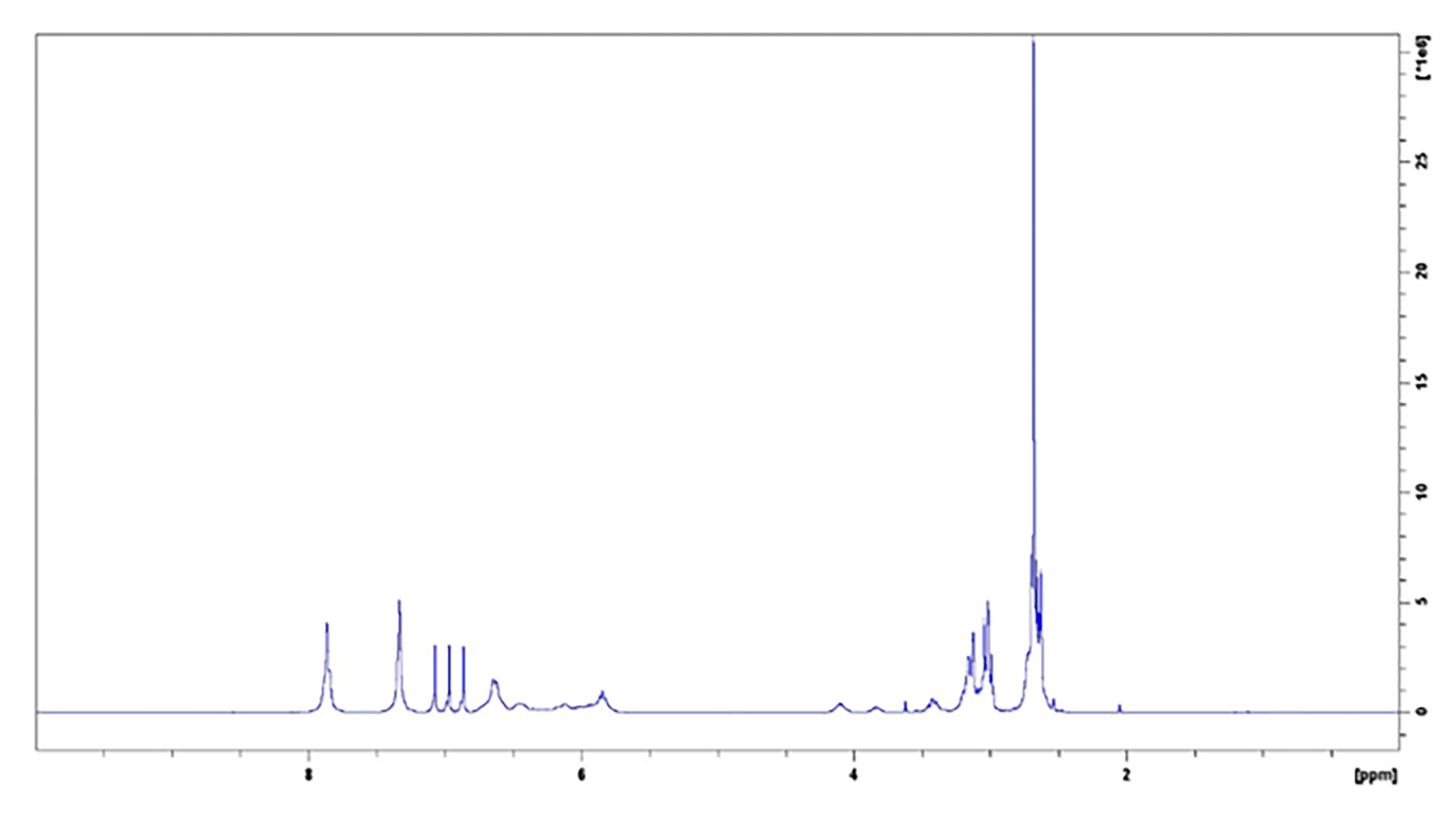


**Figure S2.** ^1^H NMR (500 MHz) for 5% D_2_O aqueous solution of 122 mM complex 4 at 293 K. Absence of the 2.1 ppm signal of ligand alone (Figure S1) suggests the S-methyl is bound to Pt, while emergence of a number of peaks between 6.8 and 5.5 ppm suggests the amino group is bound to Pt as well. Other groups, including CH, CH_2_ and amide NH_2_ groups all show different chemical shift changes.

**
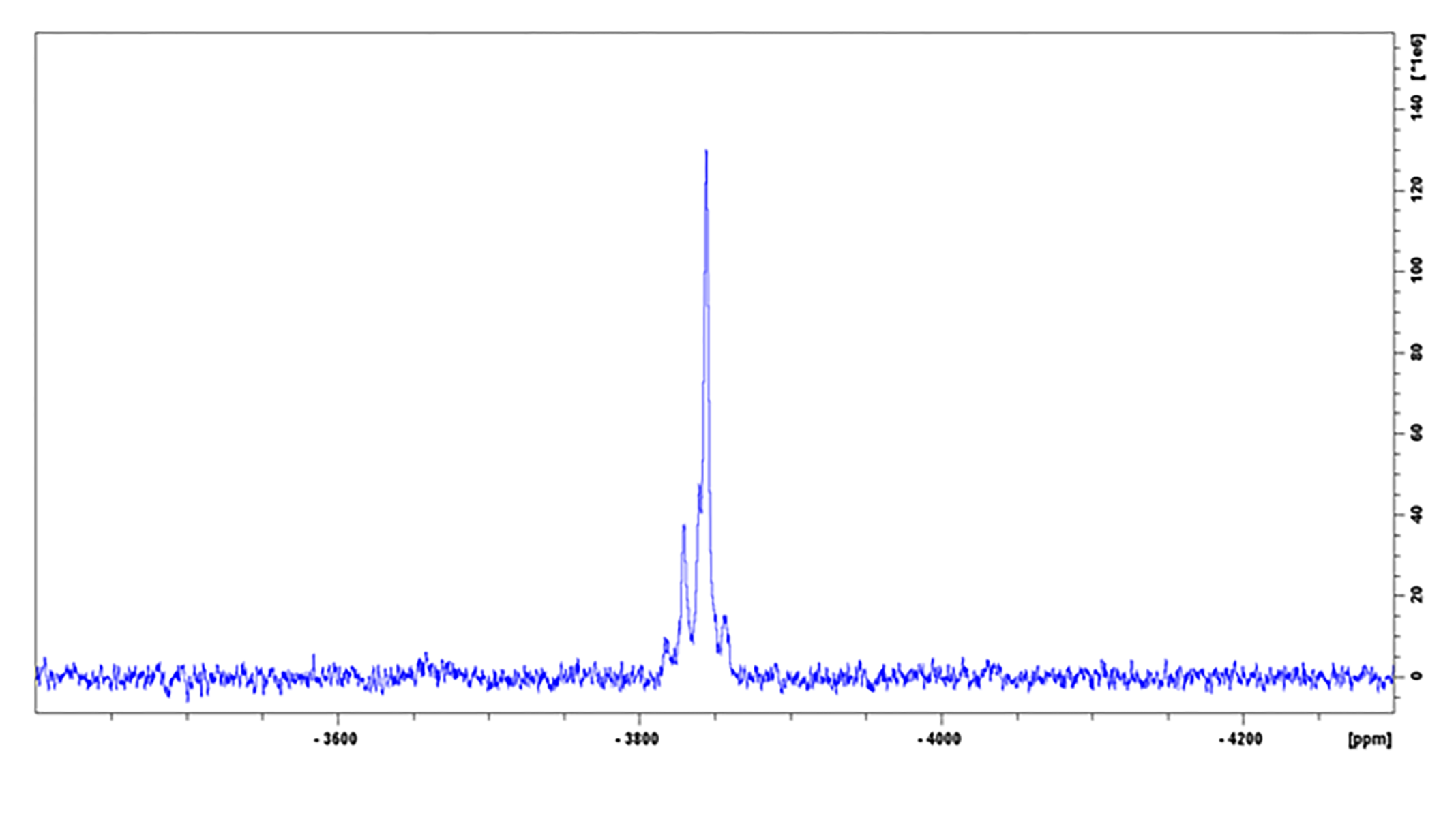
**

**Figure S3.** ^195^Pt NMR (500 MHz) for a fresh solution of complex 4 in milliQ purified water with 5% v/v D_2_O at 293 K. Broad sginals at around –3850 ppm are generally consistent with Norman et al’s report for complex 1 and similar to that of complex 3 in this study (Norman et al., 1992).


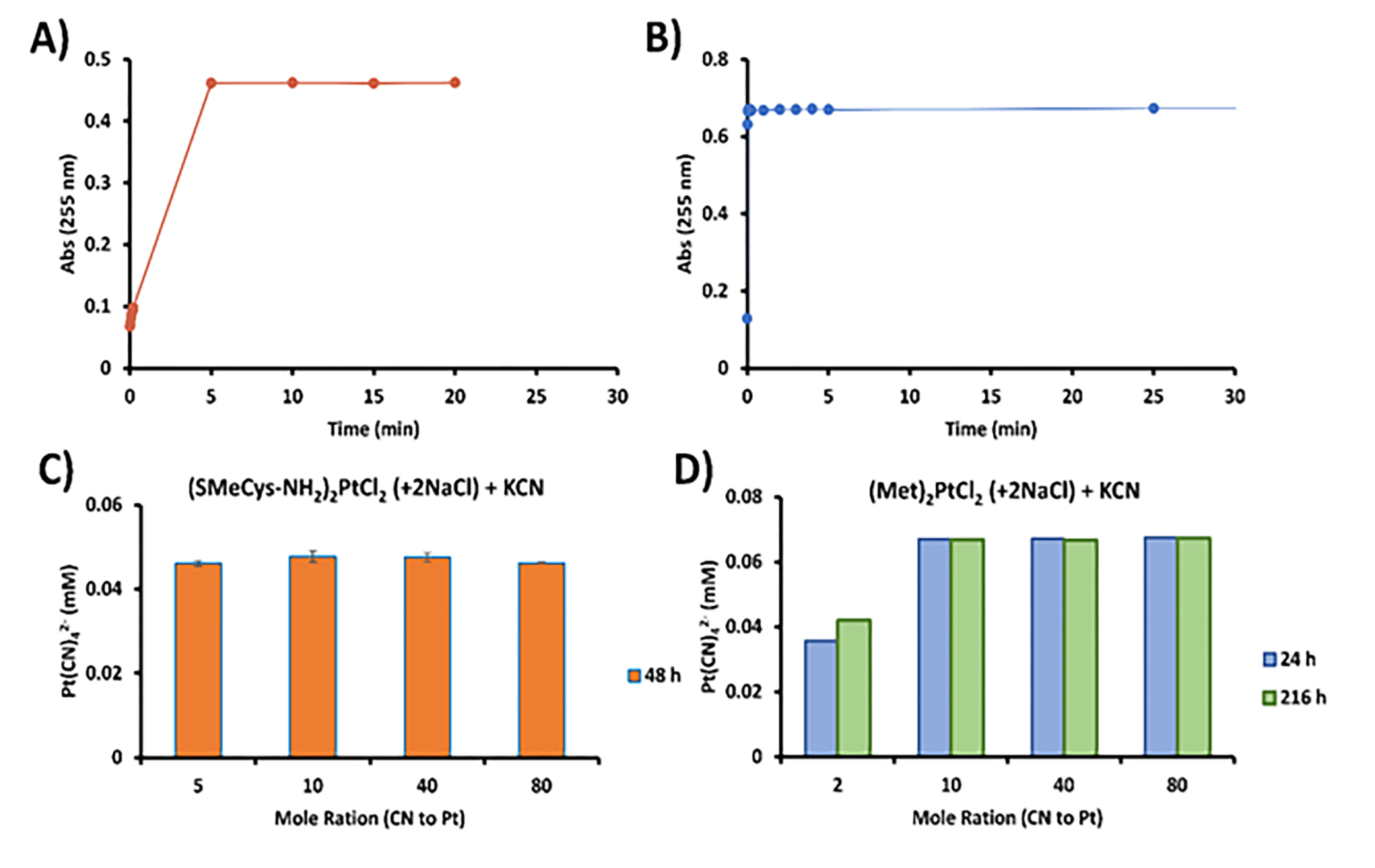


**Figure S4.** As representative examples, reaction of **A)** complex **4** (+2NaCl) or **B)** complex **1** (+2NaCl) with 1:40 Pt:CN^-^ in purified water. The samples were also incubated for a minimum of 24 h and Pt(CN)_4_^2-^ was quantified by UV-Vis once the signal was stable and consistent with Day 0 kinetics. Complex **4** (+2NaCl) and complex **1** (+2NaCl) (**C** and **D** respectively) reacted with KCN in 1:5, 1:10, 1:40, 1:80 Pt:CN in purified water. Both results demonstrate the signal is stable after 5-10 minutes of cyanide addition.


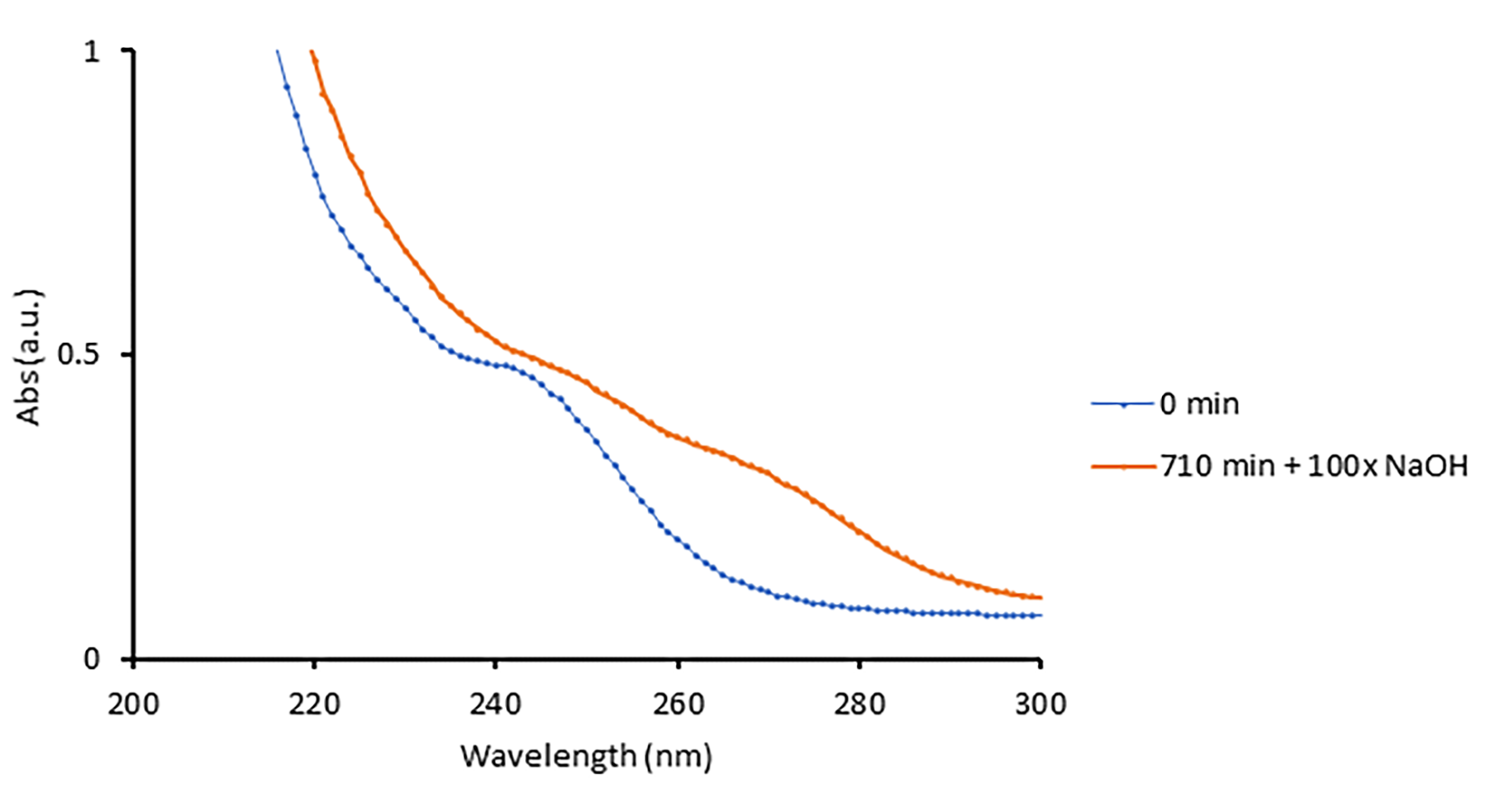


**Figure S5.** UV-Vis spectra of **4** in purified water, then adding 100 molar equivalents of NaOH to raise the pH and monitor spectral changes over 710 minutes. Molar equivalents are defined relative to the molar level of complex 4.


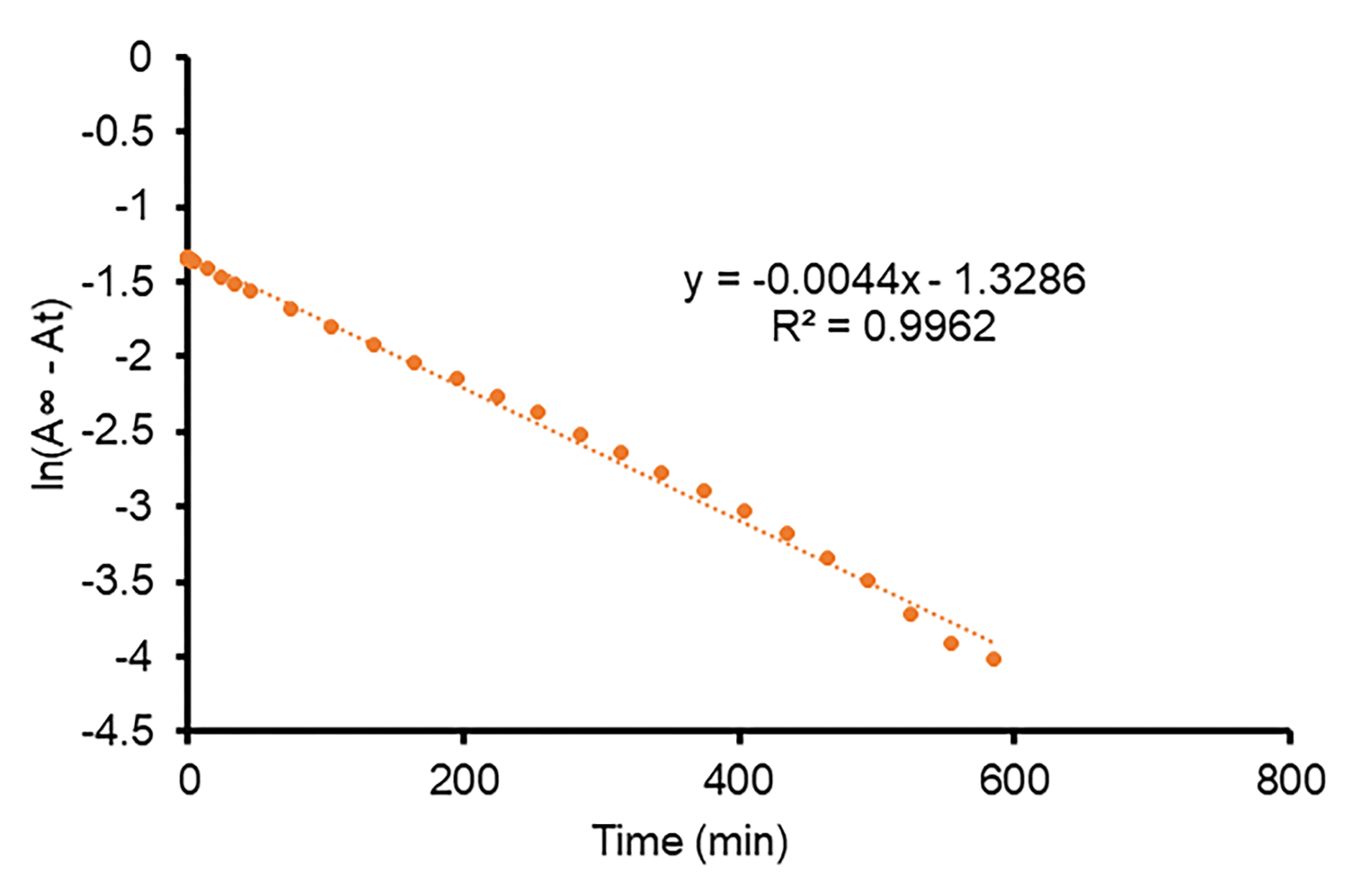


**Figure S6.** In the presence of KCN, absorption changes for complex **2** monitored at 245 nm. Complex **2** was prepared in a pH 7.26 phosphate buffer at 18.8 °C. Data shown is a result of the absorbance increase at 245 nm. Scans were taken every 0.1 min, 1 min and 30 minutes respectively over the analysis. Data fits linearly with a semi log plot suggesting the process follows first order kinetics. Half-life of appearance for these conditions is 2.6 hr with R^2^=0.9962.


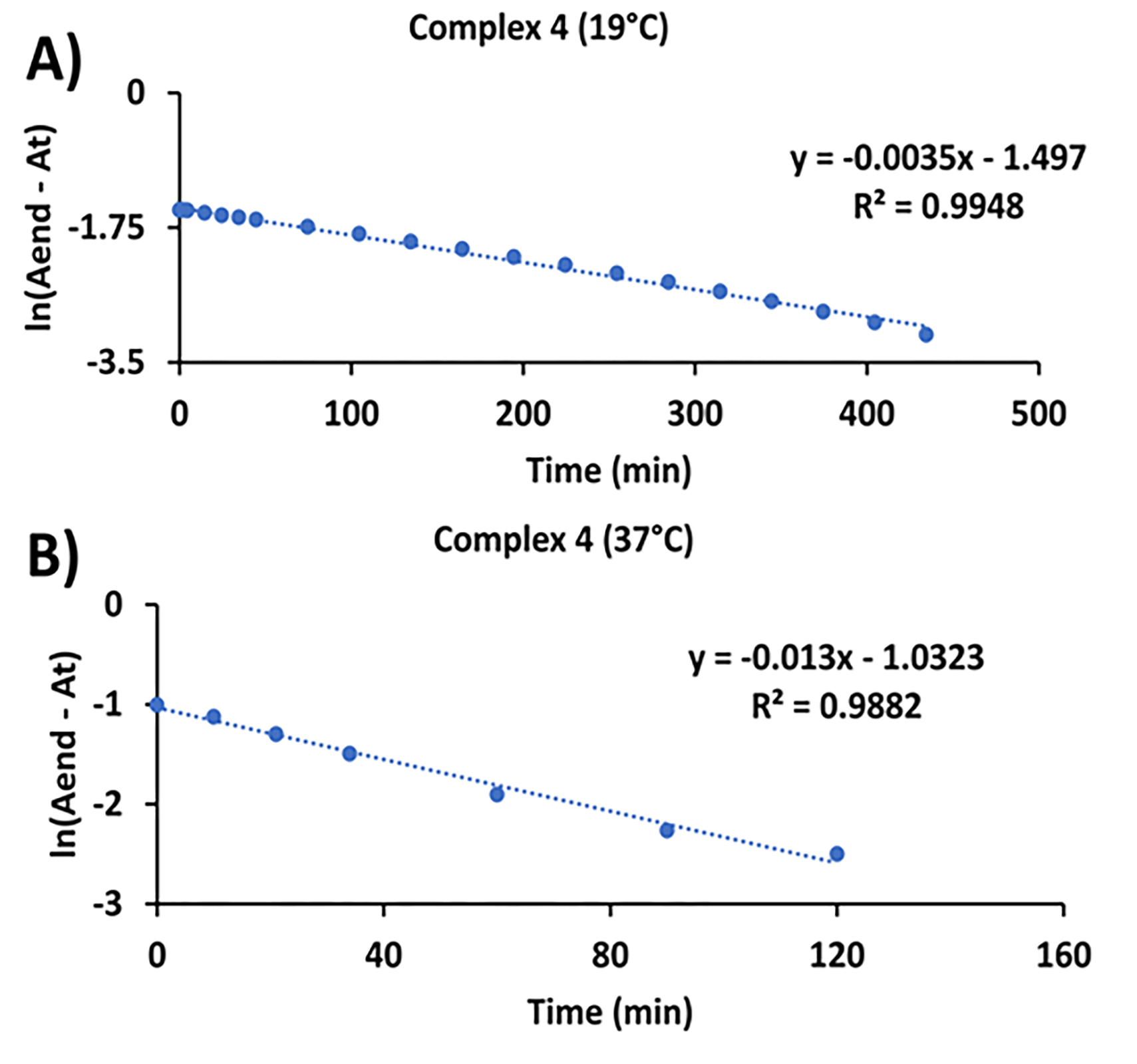


**Figure S7.** Time dependent changes of **4** in phosphate buffer pH 7.3 at 19°C (**A**) and 37°C (**B**) when allowed to react with KCN. Data were acquired by monitoring signal at 241 nm.


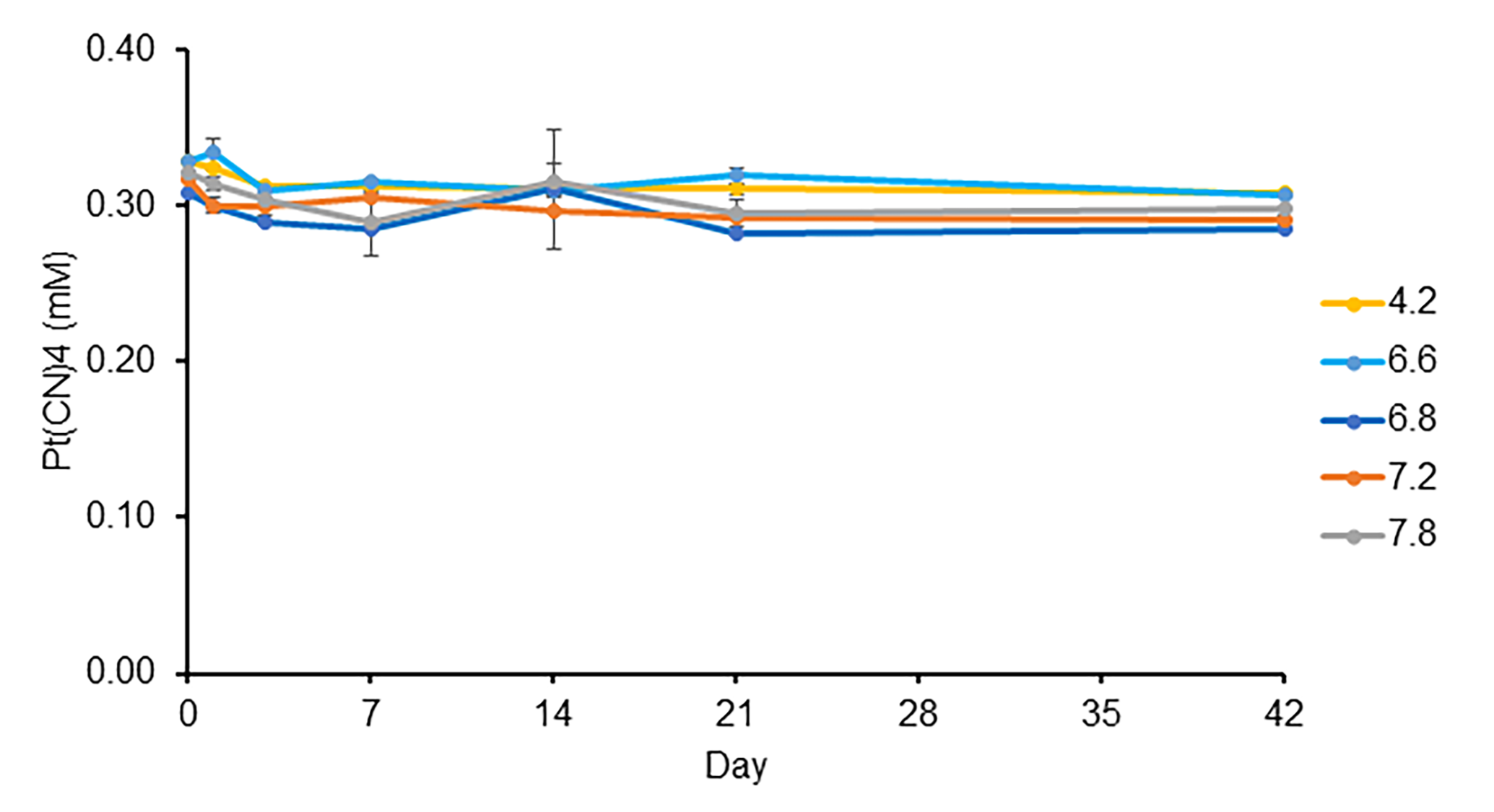


**Figure S8.** Reaction of **1** with 4 mole equivalents of potassium cyanide for 10 minutes. Reaction mixture was injected onto HPLC to quantify Pt(CN)_4_^2-^ produced to monitor reactivity for 42 days. Data shown for time point is the average of 3 replicates.


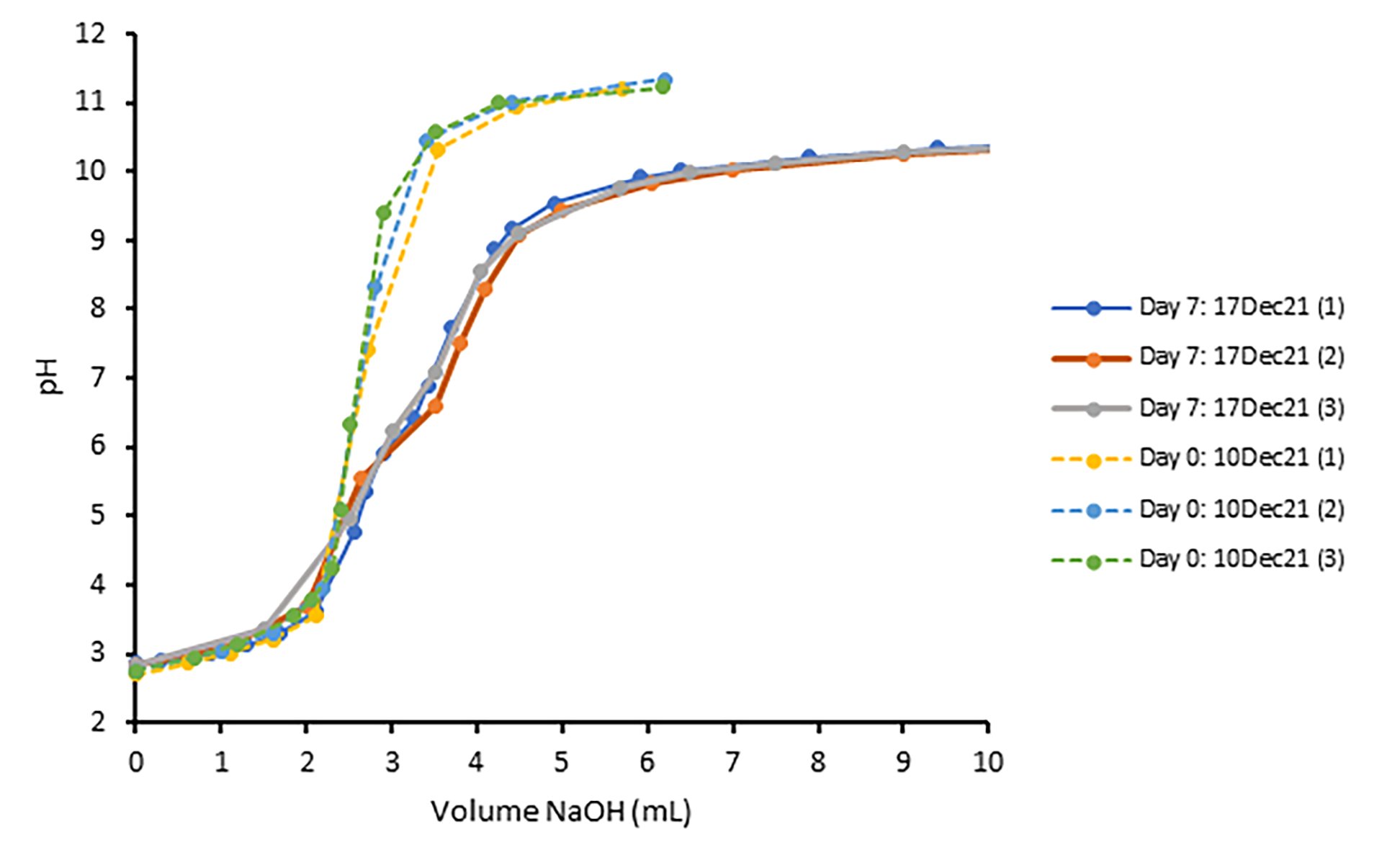


**Figure S9.** Titration of ~ 5 µmoles of **1** with 5 mM NaOH after freshly prepared in water versus aged in RT for 7 days.


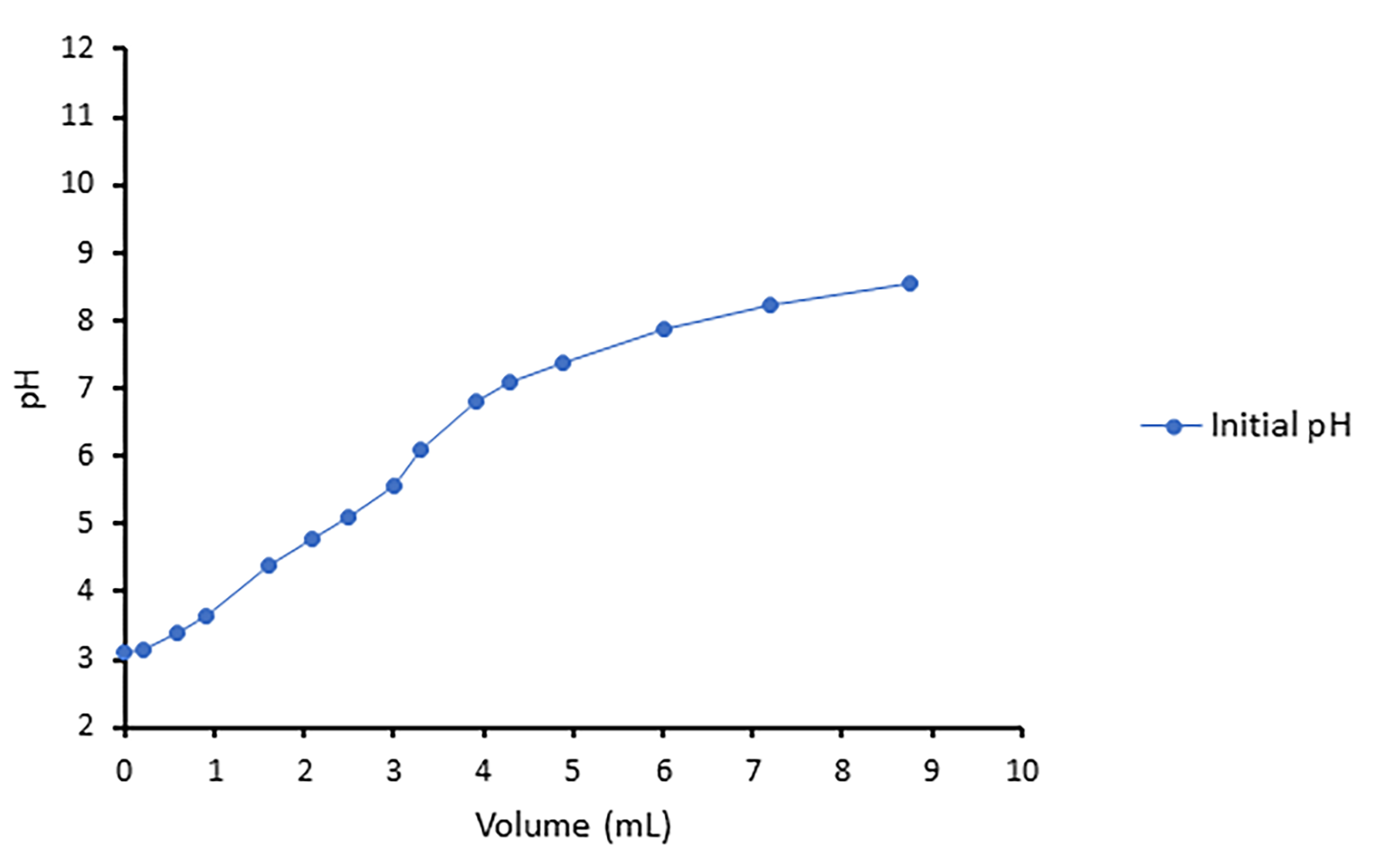


**Figure S10.** Titration of complex **2** with NaOH shows a lack of a clear equivalence point indicating a more complex chemical process than titration of ionizable group.


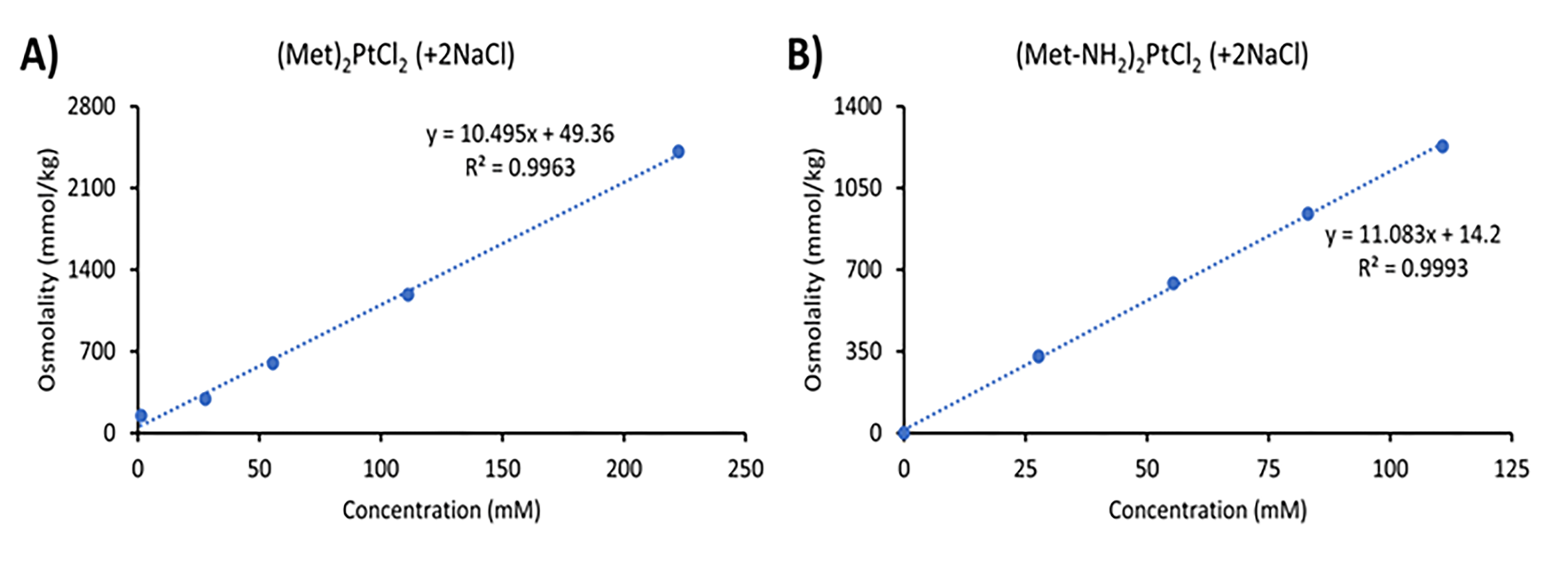
**Figure S11.** Osmolality curves for **1** (+ 2NaCl)) and **2** (+2NaCl)) **A** and **B** respectively. Data was taken from a single stock solution and subsequently diluted with purified water. Results show the osmolality decreases linearly with concentration with a correlation coefficient > 0.99.


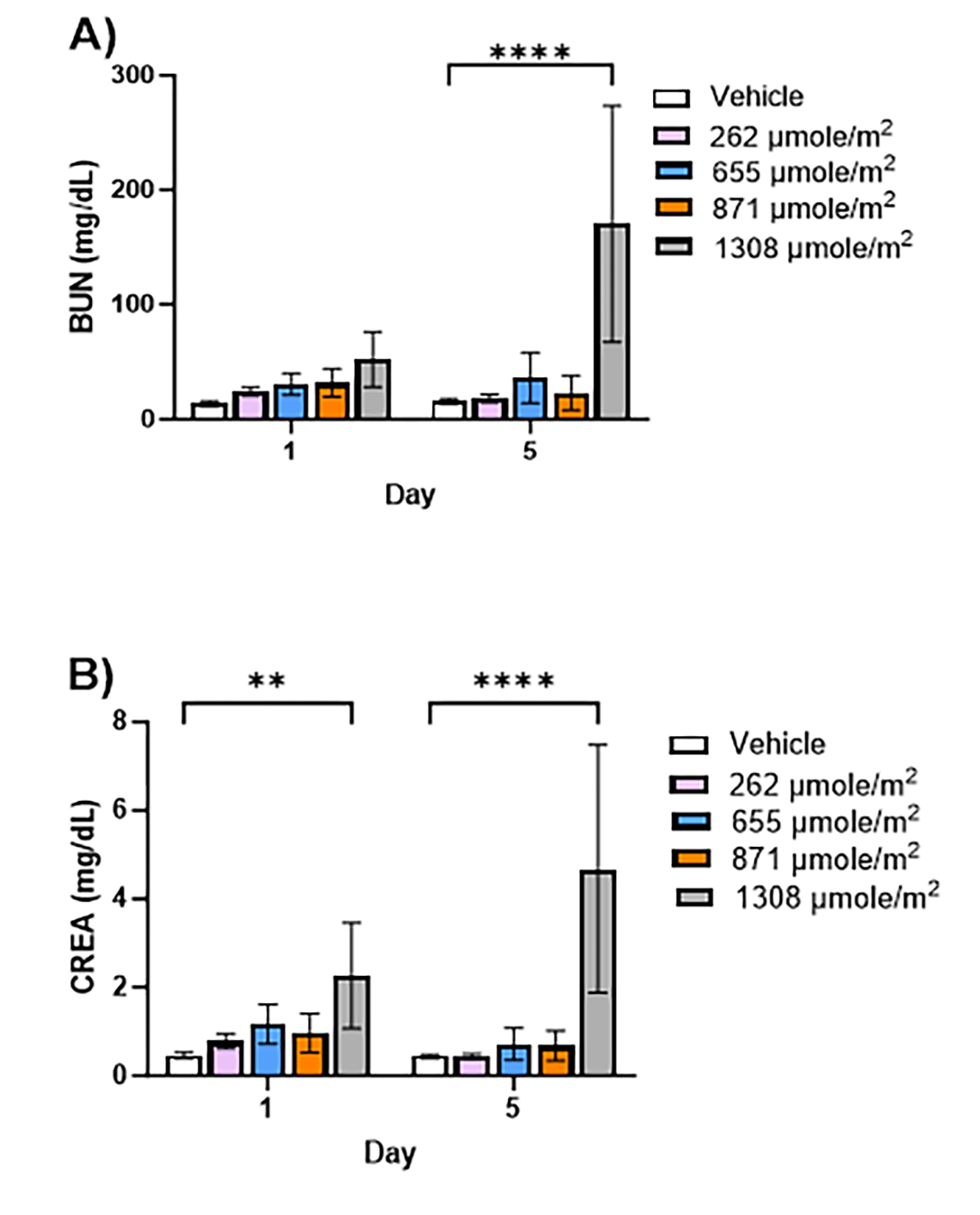


**Figure S12.** Complex **1α** dosed via IP injection. A) Mean BUN concentrations for the male and female cohort show significant (p < 0.0005) signs of AKI after 5 days at 218 µmole/kg (42.5 mg Pt/kg). B.) Mean CREA concentrations show significant levels compared to the vehicle after day 1 and 5 (p < 0.05 and p < 0.0005, respectively). Each animal received the complex in pH 6.5 phosphate buffer with an injection volume of ≤ 2.12 mL/kg. Each group 262-871 µmole/m^2^ had n=6 rats, vehicle and 1308 µmole/m^2^ contain n=12 rats. Analysis was carried out using an ordinary 2way ANOVA with a Šidák multiple comparisons comparing the cell means regardless of rows and columns.


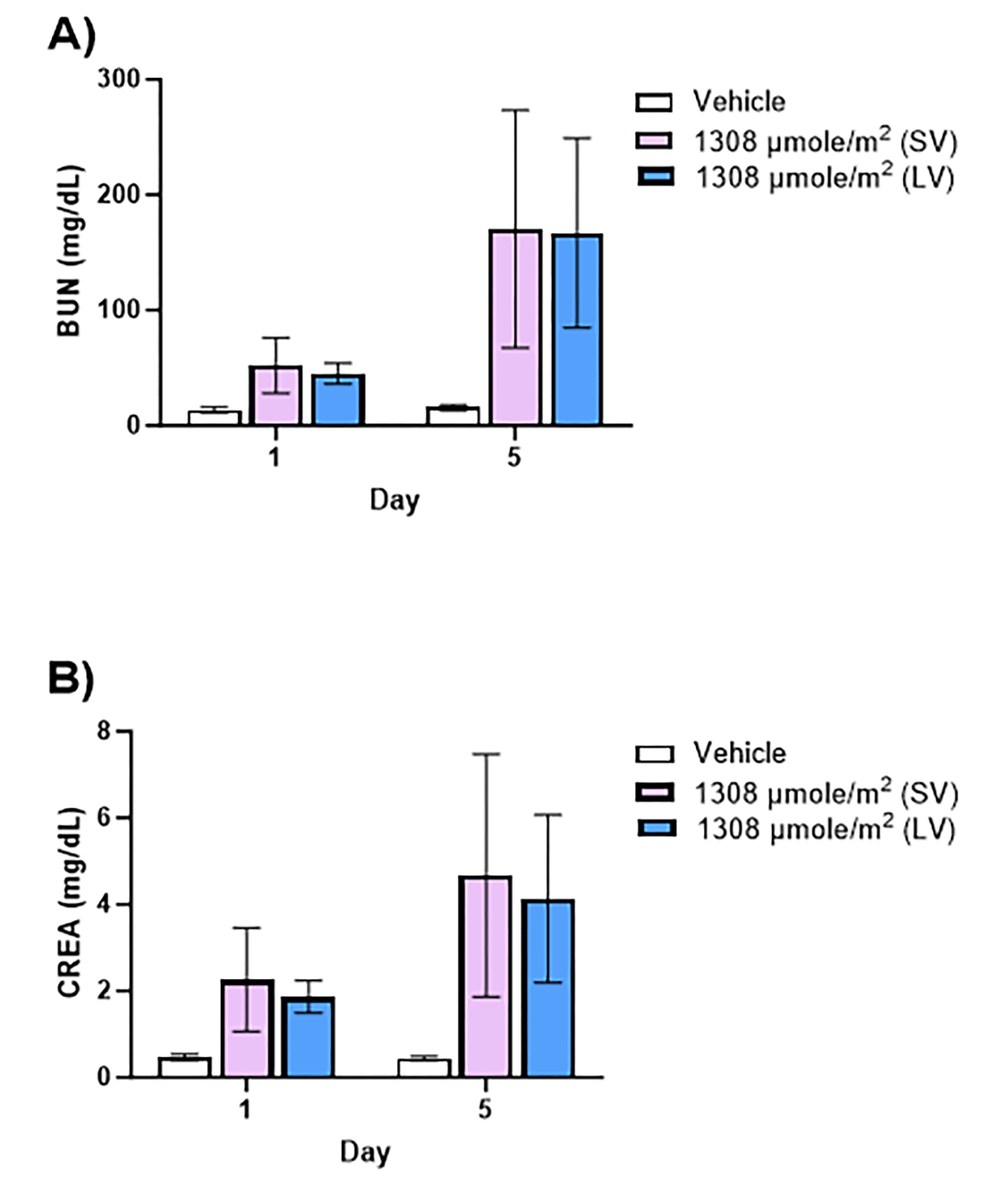


**Figure S13.** Two formulations of **1α** were compared with vehicle. Complex **1** was administered in a small volume (SV) 2.12 mL/kg n=11, and a large volume (LV) 5.28 mL/kg n=6. Analysis was carried out using an ordinary 2way ANOVA with a Šidák multiple comparisons comparing the cell means regardless of rows and columns.

**
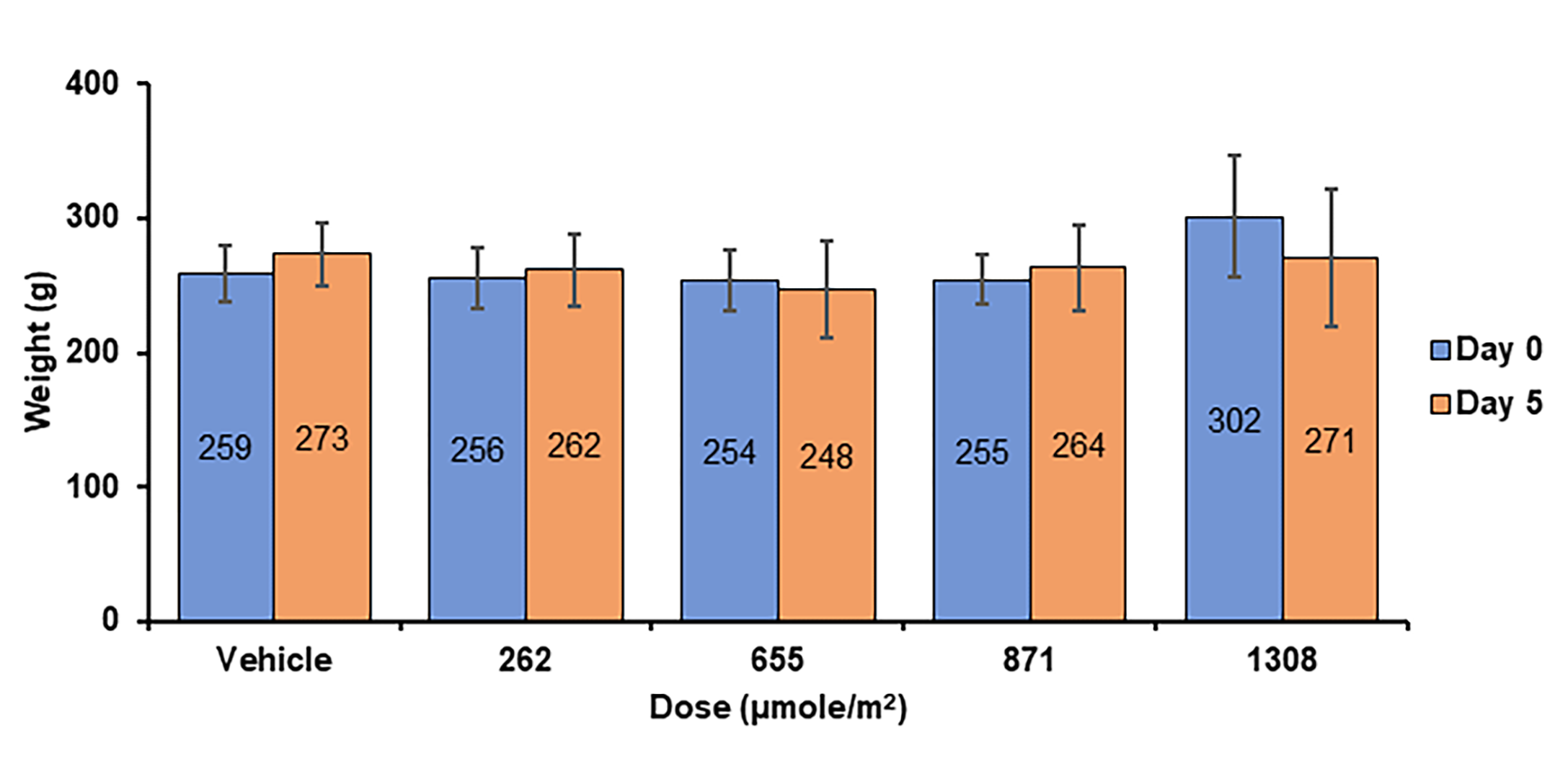
**

**Figure S14.** Body weight of rats dosed with increasing amounts of **1**. Each animal received the complex in pH 6.5 phosphate buffer with an injection volume of ≤ 2.12 mL/kg. Each group 262 – 871 µmole/m^2^ had n=6 rats, vehicle and 1308 µmole/m^2^ contain n=12 rats.


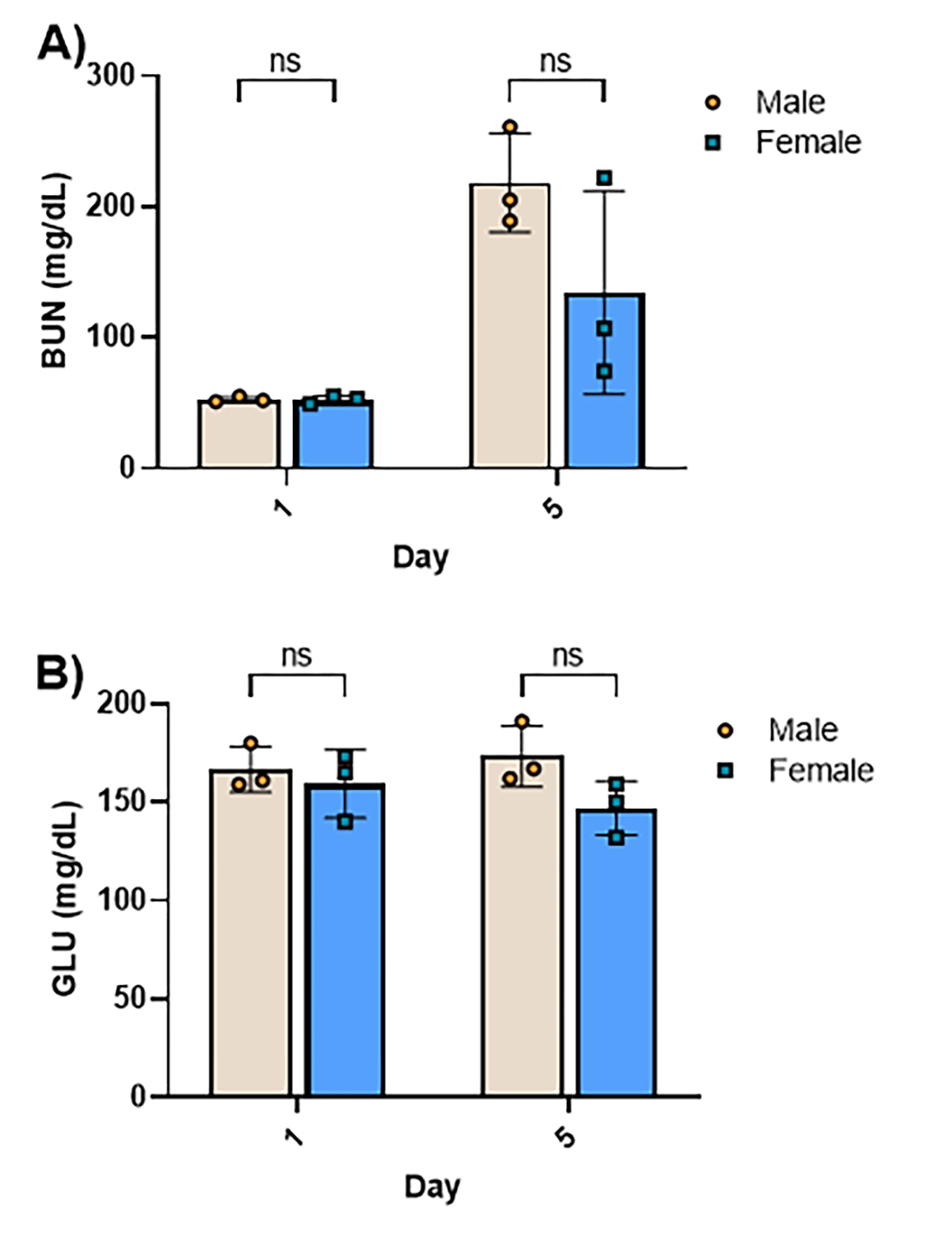


**Figure S15.** Rats were dosed with **1α** at 1308 µmole/m^2^, osmolality 1250 mmol/kg. Group size is n=3 for both female and males. BUN and GLU (**A** and **B**) are provided as example of markers of kidney dysfunction. Differences were not observed in the phosphate levels, and thus they are not shown here. This plot is provided to demonstrate a small gender difference within the study cohort. Analysis was carried out using an ordinary 2way ANOVA with a Šidák multiple comparisons comparing the cell means regardless of rows and columns.

**
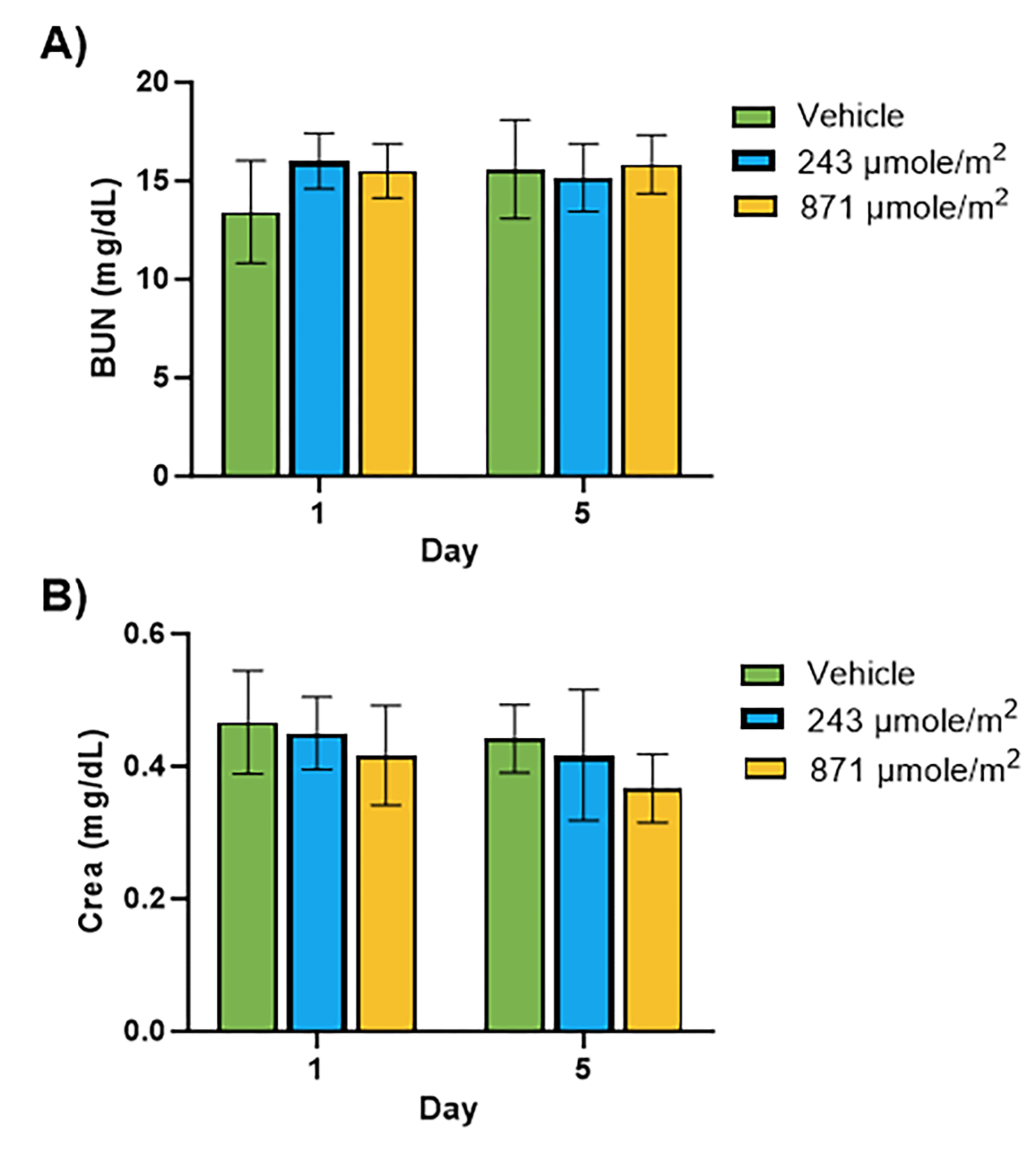
**

**Figure S16.** Rats were dosed with Pt(CN)_4_^2-^ at 243 µmole/m^2^ or 243 µmole/m^2^. Results are compared against a vehicle control containing phosphate buffer. BUN and GLU (**A** and **B**) are provided as markers for kidney dysfunction. There was no observed difference in other markers collected, and thus they are not shown here. This plot is provided to demonstrate at up 871 µmole/m^2^ no significant renal toxicity was observed after 5 days. Analysis was carried out using an ordinary 2way ANOVA with a Šidák multiple comparisons comparing the cell means regardless of rows and columns.
